# Graph-informed convolutional autoencoder to classify brain responses during sleep

**DOI:** 10.3389/fnins.2025.1525417

**Published:** 2025-04-28

**Authors:** Sahar Zakeri, Somayeh Makouei, Sebelan Danishvar

**Affiliations:** ^1^Faculty of Electrical and Computer Engineering, University of Tabriz, Tabriz, Iran; ^2^College of Engineering, Design and Physical Sciences, Brunel University London, Uxbridge, United Kingdom

**Keywords:** auditory stimuli, convolutional neural network, EEG, functional connectivity, graphical representation, sleep

## Abstract

Automated machine-learning algorithms that analyze biomedical signals have been used to identify sleep patterns and health issues. However, their performance is often suboptimal, especially when dealing with imbalanced datasets. In this paper, we present a robust sleep state (SlS) classification algorithm utilizing electroencephalogram (EEG) signals. To this aim, we pre-processed EEG recordings from 33 healthy subjects. Then, functional connectivity features and recurrence quantification analysis were extracted from sub-bands. The graphical representation was calculated from phase locking value, coherence, and phase-amplitude coupling. Statistical analysis was used to select features with *p*-values of less than 0.05. These features were compared between four states: wakefulness, non-rapid eye movement (NREM) sleep, rapid eye movement (REM) sleep during presenting auditory stimuli, and REM sleep without stimuli. Eighteen types of different stimuli including instrumental and natural sounds were presented to participants during REM. The selected significant features were used to train a novel deep-learning classifiers. We designed a graph-informed convolutional autoencoder called GICA to extract high-level features from the functional connectivity features. Furthermore, an attention layer based on recurrence rate features extracted from EEGs was incorporated into the GICA classifier to enhance the dynamic ability of the model. The proposed model was assessed by comparing it to baseline systems in the literature. The accuracy of the SlS-GICA classifier is 99.92% on the significant feature set. This achievement could be considered in real-time and automatic applications to develop new therapeutic strategies for sleep-related disorders.

## 1 Introduction

Sleep has an important role in maintaining physical and mental health (Lange et al., 2010; Xie et al., 2013; Stein et al., 2008; Kaneita et al., 2009). Getting a good night’s sleep has numerous benefits, including improved memory, creativity, concentration, and reduced stress and fatigue. However, sleep disorders are becoming increasingly common, particularly among the aging population (Ohayon et al., 2004) and suffering from psychiatric disorders (Melo et al., 2016; Yao et al., 2024). Approximately 50–70 million adults in the United States alone struggle with sleep difficulties (Medicom MTD, 1997). While pharmaceutical sleep aids help alleviate poor sleep (Yue et al., 2023), they often come with a range of negative side effects, the risk of addiction over time, and the high cost associated with prescriptions (Ford et al., 2014). To address these issues, researchers need to explore non-pharmacological tools that are cost-effective and support healthy sleep.

Music therapy is broadly utilized as a non-pharmacological method for enhancing sleep quality (Su et al., 2013; Lai and Good, 2006; Chen et al., 2021). In recent years, several studies have been conducted to examine the beneficial effects of music on human sleep quality (Truong et al., 2020; Huang et al., 2018; Gao et al., 2020; Cordi et al., 2019; Trahan et al., 2018). For example, Jespersen et al. (2015) conducted six studies involving 314 patients with insomnia, which demonstrated that music improved subjective sleep quality. Similarly, sedative music was found to subjectively improve sleep quality in patients with sleep complaints (De Niet et al., 2009). Researchers have extensively examined the function of the human brain during sleep and have found that it remains active homologous to its activities during wakefulness.

Electroencephalography (EEG) as a technique in cognitive neuroscience research, allows for real-time measurements of changes in cortical activity associated with music listening, even during different stages of sleep (Truong et al., 2020). Over the past decade, several methods have been developed to investigate brain activity during different sleep phases (Rechichi et al., 2021; Wen, 2021; Lin et al., 2017). Researchers (D'Atri et al., 2021; Gorgoni et al., 2016; De Gennaro et al., 2007) have reported a remarkable decrease in sleep spindles and K-complexes during non-rapid eye movement (NREM) sleep in patients with Alzheimer’s disease. Furthermore, initial observations during rapid eye movement (REM) sleep indicate an increase in low-frequency rhythms accompanied by a decrease in high-frequency rhythms, similar to those observed in wakefulness EEG patterns (Brayet et al., 2016). Other studies have demonstrated that analyzing the characteristics of both music and EEG reveals the potential of brain-wave music in certain clinical enhancement symptoms, such as pain (Hunt et al., 2021; Dileo, 1999; Hauck et al., 2013).

Phase-amplitude coupling (PAC) has been demonstrated to play a role in various cognitive processes, such as attention, working memory, language, and intelligence (Sacks et al., 2021). Recent evidence has shown that PAC is linked to mental health and cognition, both during periods of rest and before and after interventions (Sacks et al., 2021). This indicates that PAC possesses enduring and consistent aspects making it suitable for longitudinal studies. The emerging research suggesting that PAC could be a significant biomarker for mental health underscores the need for longitudinal research that examines PAC throughout the rapid and varying structural changes during adolescence (Dong et al., 2022). In this work, dynamic recurrence analysis (RQA) is performed to derive useful dynamic attributes from the various states of the EEG signal (Baghdadi et al., 2021). Several studies have employed RQA to quantify cortical function at different sleep stages (Rolink et al., 2015), epileptic disorder (Acharya et al., 2011), tactile roughness discrimination (Baghdadi et al., 2021), and sleep apnea syndrome (Heunis et al., 2018). It has the capability to extract complex characteristics of the signal and deterministic behavior of EEGs.

Machine learning (ML) and deep learning (DL) algorithms are highly profitable in constructing automated pipelines for detecting neurological conditions and cognitive behavior, particularly in scenarios with large volumes of data (Vu et al., 2018; Badrulhisham et al., 2024). Automatic sleep stages classification methods include support vector machine (SVM) (Wen, 2021; Gurrala et al., 2021; Lajnef et al., 2015), random forest (RF) classifier (Fraiwan et al., 2012), artificial neural networks (ANN) (Aydoğan et al., 2015), recurrent neural networks (RNNs) (Michielli et al., 2019), convolutional neural networks (CNNs) (Khalili and Asl, 2021; Hu et al., 2024; Mostafaei et al., 2024; Ma et al., 2023), and gated recurrent unit (GRU) (Moctezuma et al., 2024). Some studies utilize feature-based approaches (Basha et al., 2021; Kim et al., 2020), while more recent ones have concentrated on DL approaches (Jadhav et al., 2020; Zhu et al., 2020) employing data from one or two EEG channels. Nevertheless, the use of handcrafted features offers advantages such as interpretability and domain-specific insights, which can improve model performance, especially in situations with limited data or where domain knowledge is crucial. However, a key challenge is that handcrafted features may not generalize well across diverse tasks, unlike deep learning techniques that can automatically learn relevant features from raw data. For example, in EEG analysis, handcrafted features like phase locking value (PLV) and coherence provide physiologically meaningful insights, but their effectiveness may vary across different classification tasks or datasets.

In this study, EEG signals of healthy subjects were recorded during four different states: wakefulness, NREM sleep, REM sleep with the representation of 18 different stimuli, and REM sleep without any stimuli. After preprocessing and feature extraction, significant features were converted into graph representations to extract deep spatial features. Simultaneously, RQA was employed to clean EEG signals and capture the dynamic changes in brain activity. Then, graphical representations of the selected features were fed to an attention-based convolutional autoencoder neural network. This model incorporates dynamic neural connectivity topologies such as PLV, PAC, which were converted to graph signals and modified by applying the RQA on the attention layer of the CNN. This approach aids in capturing spatial and temporal features for high-resolution dynamic functional connectivity discovery that advances accurate sleep phase detection. We propose a cutting-edge deep learning model, known as graph-informed convolutional autoencoder (GICA), to classify four sleep stages, called “SlS” and 18 states of brain responses to stimuli during the REM sleep phases, called “BRSl.” Precisely classifying sleep states is crucial for diagnosing and monitoring sleep disorders, providing valuable insights into individuals’ sleep patterns and overall wellbeing. In other words, detecting brain responses to various types of music can be used as non-pharmacological therapy for sleep disorders to introduce individual music therapy methods and reduce invasive protocols.

The organization of this paper is outlined as follows: Section 2 provides an overview of the dataset employed in this research followed by statistical analyzer methods and a novel feature extraction process. Section 3 presents the simulation results and visualization of the brain function during sleep and various types of music stimuli. Finally, we conclude our study and outline avenues for future research in Section 4.

## 2 Materials and methods

**Figure 1:**
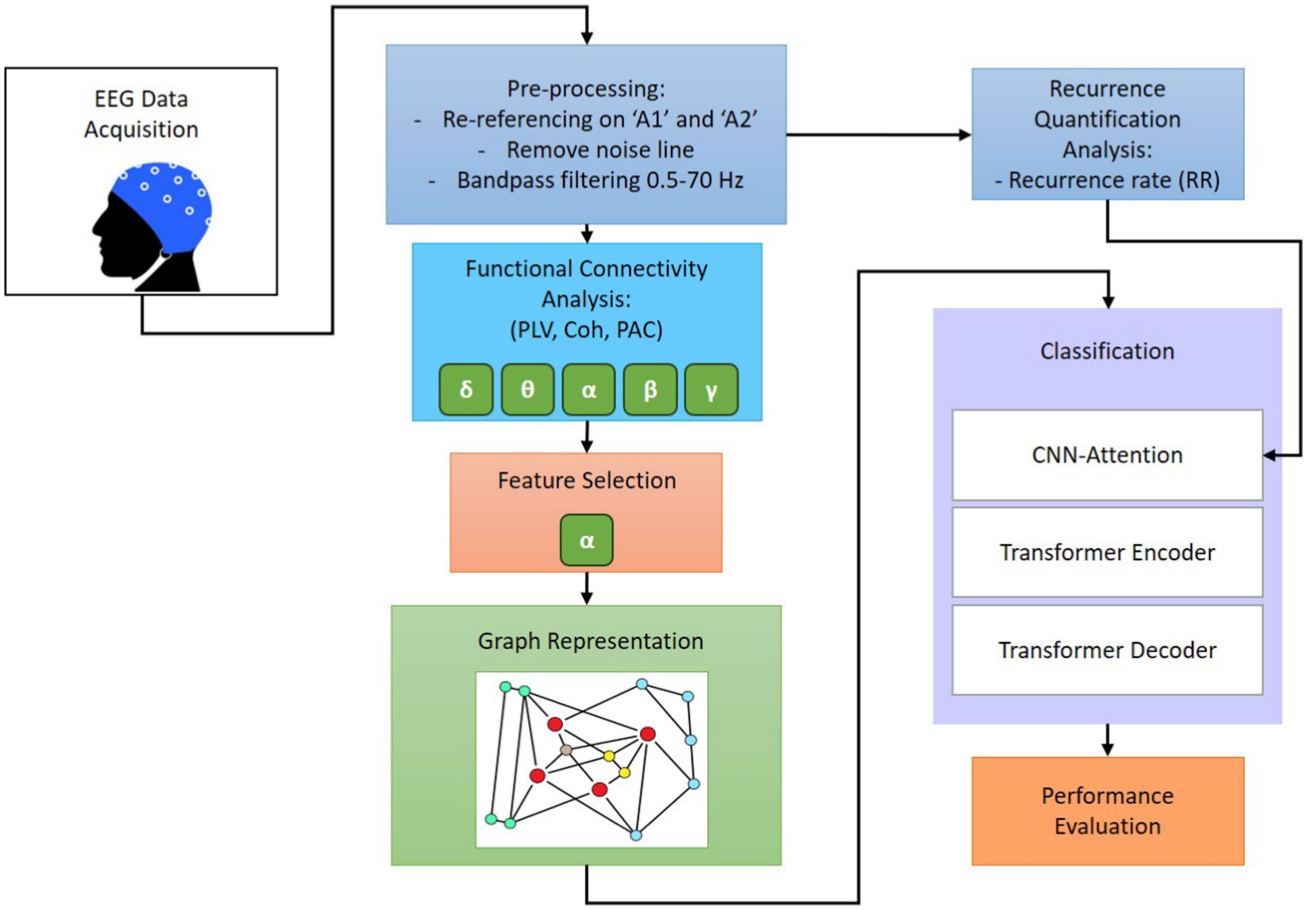
Schematic representation of the proposed GICA framework.

This paper is intended to improve machine learning-based sleep stage detection by implementing six main steps: preprocessing, feature extraction, feature selection, graph representation, dynamic analysis, and classification. The focus of this study is to investigate functional connectivity, graph generation, and RQA to determine a robust feature set that provides insights into information from multiple domains. Significant features were combined to illustrate the changes in wakefulness, NREM, REM sleep with stimuli, and REM sleep without stimuli over the brain regions. To achieve satisfactory classification performance, this research conducts a comparative study to evaluate the effect of individual PLV, coherence, PAC, and RR features on various EEG sub-bands, as well as the combined significant graph-based feature set. Consequently, the GICA framework is identified according to the graph representation of selected features and RQA to enhance the classification performance of the convolutional autoencoder. The proposed GICA framework is validated using a multi-channel EEG dataset in two categories: first, to classify four different stages including wakefulness, NREM sleep, REM sleep with stimuli, and REM sleep without stimuli, called SlS; second, to classify brain responses to 18 different stimuli during REM sleep, called BRSl. Figure 1 shows the schematic block diagram of the proposed GICA algorithm. The goal is to determine whether distinct neural responses to these stimuli could be differentiated using machine learning.

### 2.1 Participants

The present study was conducted at the Biomedical Engineering Laboratory of the University of Tabriz in Iran. To examine the impact of sound stimuli on sleep, a group of healthy participants with a consistent habit of staying up late were selected for the experiment. A total of 36 subjects (33 right-handed and 3 left-handed) were recruited who had no history of psychological disorders or medication use. Three participants woke up during the experiment and were subsequently excluded from further analysis. The data from the remaining 33 subjects (19 females; mean age = 31.06 ± 13.75 years) were included in the final analysis. Before their participation, all subjects provided informed consent and completed a health questionnaire.

### 2.2 Data recording protocol

According to the guidelines of the American Academy of Sleep Medicine (AASM) (Khalighi et al., 2016), sleep is categorized into five stages: wakefulness (Wake), non-rapid eye movement (NREM) sleep, which consists of three substages (N1, N2, and N3), and rapid-eye movement (REM) sleep. In this study, EEG recordings were collected during four main states:

- State 1 (wakefulness—EO): 5-min recording while subjects were lying down with eyes open (EO),
- State 2 (NREM sleep—EC): 5-min recording during lying down with eyes closed (EC) and transition into NREM sleep,
- State 3 (REM sleep with stimuli—SlpWiSt): 20-min recording during REM sleep with auditory stimuli presentation,
- State 4 (REM sleep without stimuli—SlpWoSt): 5-min recording during REM sleep without auditory stimuli.

To distinguish wakefulness, NREM, and REM stages, we utilized EEG frequency band characteristics according to the AASM guidelines. The wakefulness stage exhibits dominant alpha band activity in the occipital region. Alpha rhythm (8–13 Hz) predominantly in the occipital region and/or low-amplitude, mixed-frequency activity are cues of this stage. The NREM stage is characterized by low alpha activity, theta activity, sleep spindles (12–16 Hz), and delta waves (0.5–4 Hz), which indicate the transition from shallow to deep sleep (Garcia-Molina et al., 2018). The REM stage is distinguished by low-voltage, mixed-frequency EEG activity, rapid eye movement, and low muscle tone with dominant theta waves and desynchronized EEG patterns.

Participants were instructed to lie down comfortably on a bed in a sound attenuated, temperature-controlled (~25°C), and dimly lit room (<50 Lux). No pharmacological agents were administered to induce sleep, ensuring natural sleep cycles. EEG recordings were controlled approximately 6 h per session, with each participant undergoing two separate sessions, either in the morning or afternoon, to maintain consistency. To facilitate sleep, mindfulness meditation techniques were utilized, which included minimizing distractions, lying in a comfortable position, and focusing on breathing (inhale for a count of 10, hold, exhale for a count of 10, and repeat this cycle 10 times).

Sleep stages were annotated offline by a trained sleep expert following AASM criteria. EEG signals from frontal central, and occipital electrodes were used for sleep staging. Annotations were performed after data collection, not in real-time, to ensure accurate labeling of REM sleep epochs. Electrooculogram (EOG) signal was used in the eye movement staging process.

**Figure 2:**
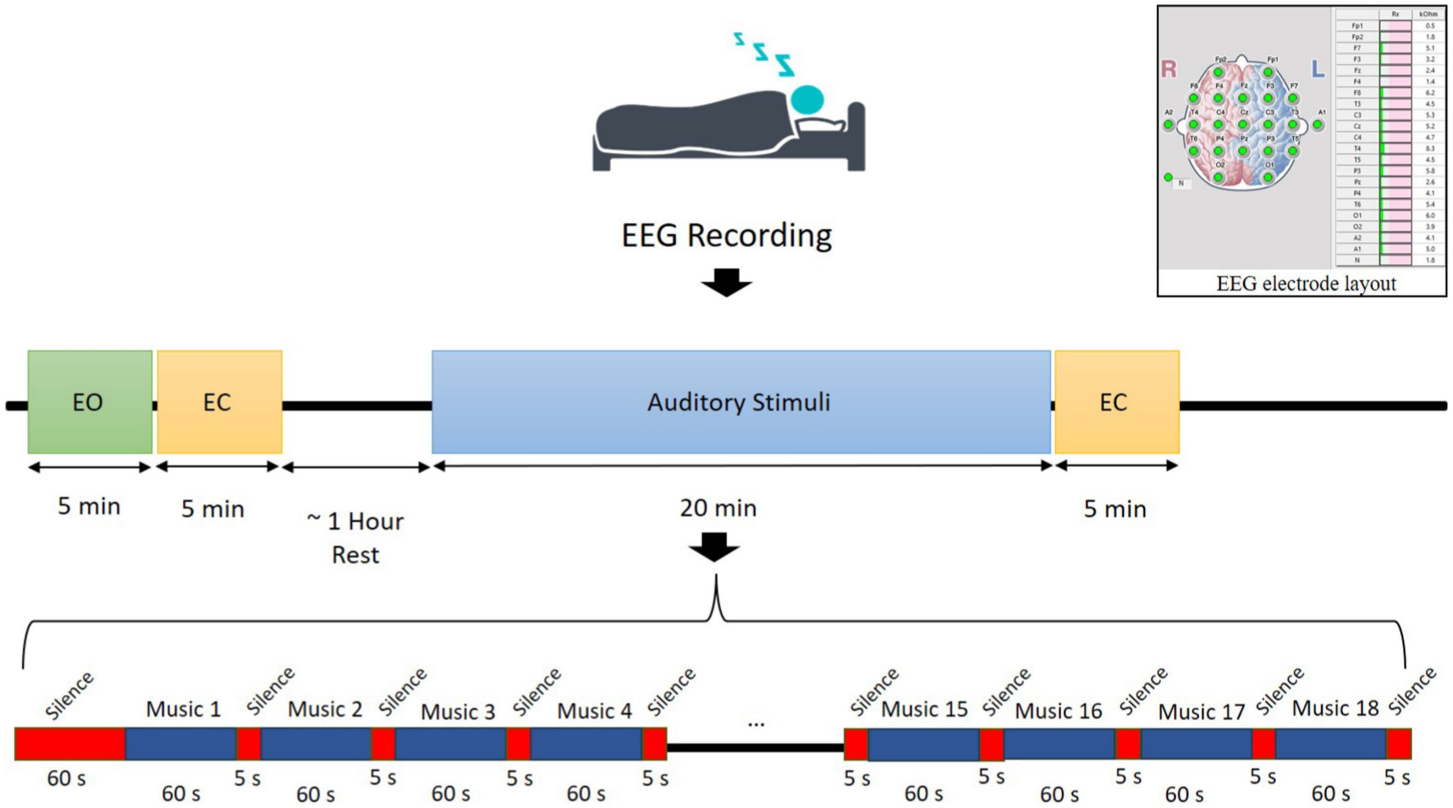
Illustration of the experimental procedure with EEG electrode layout.

Eighteen types of auditory stimuli were selected to present for participants during their sleep. These sounds include various types of instrumental and natural sounds such as piano, hang drum, guitar, saxophone, storm, rain in the forest, birds singing, ocean waves, fire crackling, whale, santoor, kamancheh, tar, and violon. Each auditory stimulus was presented only during the REM sleep to prevent habituation. Auditory stimulation was delivered using in-ear earbuds (Apple, 3.5 mm headphone plug) to ensure optimal auditory delivery during sleep. These earbuds were selected for their comfortable fit and high-fidelity sound quality, minimizing external noise interference and ensuring consistent auditory stimulation. The order of presentation was fixed across participants, with a 60-s silence before the first stimulus, followed by 5-s silence intervals between trails. Each sound was played for 60-s, leading to a total presentation duration of 1,080-s. The stimuli were presented binaurally at 45 dB SPL, a level chosen to minimize the risk of sleep disturbances while still producing measurable effects on sleep patterns. Sleep disturbance was defined as significant interruptions in sleep continuity or architecture, including arousals (transient shifts in EEG frequency lasting more than 3-s), abrupt changes in sleep stages, or prolonged wakefulness. The 45 dB SPL intensity was selected based on prior literature demonstrating that sound levels below 50 dB SPL are unlikely to cause significant sleep disruptions (Feige et al., 2021; Rudzik et al., 2018), as well as pilot data confirming that this intensity produced detectable changes in sleep patterns without increasing arousal frequency or wakefulness. Wave Pad Sound Editor^1^ was used to control the loudness and presentation order of the stimuli. The experimental paradigm is illustrated in Figure 2.

Auditory stimuli were exclusively presented during REM sleep due to its strong association with vivid dreaming and heightened brain activity in sensory processing regions. Research indicates that REM sleep is crucial for memory consolidation, particularly for emotional and procedural learning (Payne and Nadel, 2004; Genzel et al., 2015). By presenting auditory stimuli during REM, we aimed to explore how external sensory inputs are integrated into brain activity, possibly influencing dream content. Furthermore, the brain’s responsiveness to external stimuli during REM sleep is high, allowing us to investigate neural processing without fully walking the subject.

The auditory sounds used in this study consisted of a variety of sounds, which were chosen for their calming rhythmic qualities to promote relaxation during REM sleep. Regarding frequencies, the sounds generally fell within the range conducive to relaxation. The typical frequency ranges for the sounds and instruments include piano: 27.5–4,186 Hz, hang drum: 110–880 Hz, guitar: 82–880 Hz, saxophone: 55–1,200 Hz, storm: 20–120 Hz, rain in the forest: 500–5,000 Hz, birds singing: 2,000–8,000 Hz, ocean waves: 30–300 Hz, fire crackling: 500–1,000 Hz, whale songs: 10–40 Hz, santoor: 261–1,500 Hz, kamancheh: 150–1,000 Hz, Tar: 80–900 Hz, violin: 196–2,000 Hz. Each type of sound was played for the same duration in a consistent order across subjects. Although randomization was not implemented, we ensured uniform stimulus conditions to control for variability.

In this work, auditory stimuli were presented only during the REM sleep. REM Sleep is characterized by vivid dreaming and heightened brain activity, particularly in regions associated with sensory processing, such as the visual and auditory cortices. This makes REM sleep a critical phase for investigating neural responses to sensory stimuli and their integration into dream content. Presenting stimuli during this phase allows us to explore how external auditory inputs may be integrated into the dream content or processed by the brain during heightened sensory processing. Research suggests that REM sleep plays a crucial role in memory consolidation, particularly for emotional and procedural memories (Wagner et al., 2001; Rasch and Born, 2013; Siegel, 2001). The brain during REM may be more receptive to external auditory stimuli without fully walking the subject, allowing us to examine the brain’s responsiveness without disrupting sleep continuity (Navarrete et al., 2024; Sallinen et al., 1996; Dang-Vu et al., 2010).

### 2.3 EEG data acquisition

EEG signals were recorded using 19 Ag/AgCl scalp electrodes placed according to the international 10–20 system (Homan et al., 1987) by the EEGA-21/26 “Encephalan-131-03” system (Medicom MTD, 1997). The reference electrodes were positioned at the left (A1) and right (A2) mastoids, and the ground electrode was placed at the forehead (Fpz). EEG signals were sampled at 250 Hz, and electrode impedance was kept below 10 KΩ to ensure signal quality (see Figure 2). Subjects were comfortably lying down on a bed in a quiet, temperature-controlled room. No medications or external aids were used to facilitate sleep. Sleep progression was monitored by an experienced sleep technician who visually tracked EEG waveforms to ensure the detection of different sleep stages. EEG signals were recorded for two separate sessions for each participant.

### 2.4 Pre-processing

The EEG data were analyzed using MATLAB (version 2022b) and the Brainstorm toolbox (Tadel et al., 2011). The pre-processed steps included the following:

1) Montage application: EEG signals were initially re-referenced to average mastoid reference (A1/A2) to reduce common-mode noise.
2) Artifact removal: Fast independent component analysis (FastICA) (Van et al., 2016) was applied to remove eye-blinking and artifacts detecting during wakefulness and sleep. EEG signals were visually inspected to reject segments with excessive muscle artifacts and non-physiological noise.
3) Filtering: A 50 Hz notch filter was applied to eliminate power line noise. A 0.5–70 Hz Butterworth band-pass filter (Ferdous et al., 2016) was applied to remove low-frequency drifts and high-frequency artifacts.
4) Segmentation data for analysis: EEG data were segmented into 2-s non-overlapping windows to capture rapid fluctuations in brain activity, ensuring high temporal resolution. This segmentation approach is particularly effective for monitoring transitions between different sleep stages.
5) Feature extraction: after pre-processing, brain network connectivity features were computed from the clean EEG signals, including PLV, Coherence, PAC, and RQA.

**Figure 3:**
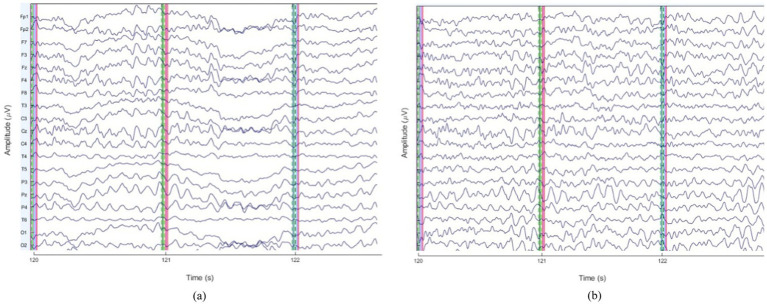
Comparison of EEG patterns: **(a)** before and **(b)** after preprocessing.

To illustrate the impact of preprocessing, Figure 3 presents sample EEG time series before and after artifact removal for a representative subject. These figures demonstrate the effectiveness of ICA-based artifact rejection and band-pass filtering in improving signal quality.

**Figure 4:**
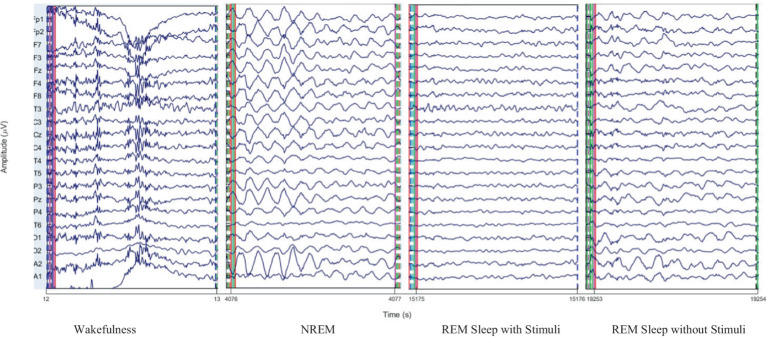
EEG signals for 1 s of wakefulness, NREM, REM sleep with and without stimuli stages.

To illustrate the nature of EEG data across different sleep stages, sample EEG waveforms for wakefulness, NREM, and REM sleep are presented in Figure 4. These EEG traces represent clean epochs recorded from 21 electrodes and showcase distinct patterns characteristic each stage. Additionally, we provide visualizations of extracted features, including PAC in the alpha and gamma bands, which utilized in our deep learning model. These insights elucidate the complexity of sleep classification and the distinct signal characteristics employed for feature learning.

### 2.5 Feature extraction

To prepare the information for the GICA-based classifier to discriminate sleep phases, functional connectivity, and dynamic state features are extracted from EEG epochs and then, converted into graph representations. Each graph representation is composed of three elements: EEG channels referred to as nodes, connection among the nodes referred to as edges, and the sets of features extracted from EEG signals recorded at the nodes referred to as graph signals. We utilize functional connectivity and spatial distance between nodes to calculate the edges. Functional connectivity captures the functional interactions between EEG channels during the synchronized firing of neurons from different brain regions that occur during sleep. The spatial distance between nodes provides basic information about the spatial distribution of the nodes in the graph representation.

In this study, we employed PLV, coherence, and PAC measures to calculate functional connectivity maps and cross-frequency coupling between different frequencies of neural oscillation. These measures quantify the strength of phase synchronization between pairs of EEG signals recorded at different channels.

- PLV: The Hilbert transform is applied to the EEG signals to measure PLV for a pair of EEG signals (Zakeri and Geravanchizadeh, 2021). Subsequently, the instantaneous phase of each signal and the relative phase between the two signals are calculated. The phase difference between pairs of EEG channels was computed at each time point within the 2-s window. PLV is defined as follows in Equation 1 (Raeisi et al., 2022):

(1) $$PLV_{xy}=\left|\frac{1}{N}\sum_{j=0}^{N-1}e^{i\varphi_{xy}\left(i\Delta \;t\right)}\right|,$$

where $\varphi_{xy}\left(t\right)$ is the relative phase between the EEG signals from channels *x* and *y*, 1/Δt indicates the sampling frequency of the EEG signals. *N* is the number of samples in one EEG epoch (Wang et al., 2019). PLV measurements can range between 0 and 1 where 0 indicates no phase synchronization and 1 indicates complete phase synchronization.

- Coh: The Welch method with a Hanning window was used for spectral estimation (Welch, 2003). Coherence quantifies the linear time-invariant relationship between two-time series *x* and *y* at a given frequency λ , and is defined as Equation 2 (Raeisi et al., 2022):

(2) $$Coh_{xy}={\left|R_{xy}\left(\lambda \right)\right|}^{2}=\frac{{\left|f_{xy}\left(\lambda \right)\right|}^{2}}{f_{xx}\left(\lambda \right)f_{yy}\left(\lambda \right)}.$$

Here, $R_{xy}\left(\lambda \right)$ represents the complex-valued coherence of *x* and *y*, $f_{xy}\left(\lambda \right)$ , $f_{xx}\left(\lambda \right)$ , and $f_{yy}\left(\lambda \right)$ represent the cross-spectrum of *x* and *y*, the power spectrum of *x*, and the power spectrum of *y*, respectively. Coherence is a positive function that is symmetric to *x* and *y* $Coh_{xy}\left(\lambda \right)=Coh_{yx}\left(\lambda \right)$ . It can range between 0 (indicating no coherence between *x* and *y*) and 1 (indicating strong coherence between *x* and *y*) (Zhang et al., 2020).

- PAC: To calculate PAC, the clean EEG signal is transformed into a complex-valued analytic signal. Subsequently, either the phase or amplitude is extracted from this complex-valued analytic signal. All of these steps can be effectively implemented using the Brainstorm toolbox (Tadel et al., 2011). PAC was computed for each 2-s EEG segment to measure the interaction between the phase of lower frequencies and the amplitude of higher frequencies. To ensure statistical significance, we employed a bootstrap-based surrogate analysis where the phase time series was randomly shuffled 1,000 times while maintaining the amplitude time series unchanged. This generated a null distribution of PAC values for each 2-s window. Observed PAC values were then compared against the 95th percentile of the surrogate distribution (*p*_value < 0.05) to determine statistical significance. Only PAC values exceeding this threshold were retained for further analysis.

**Figure 5:**
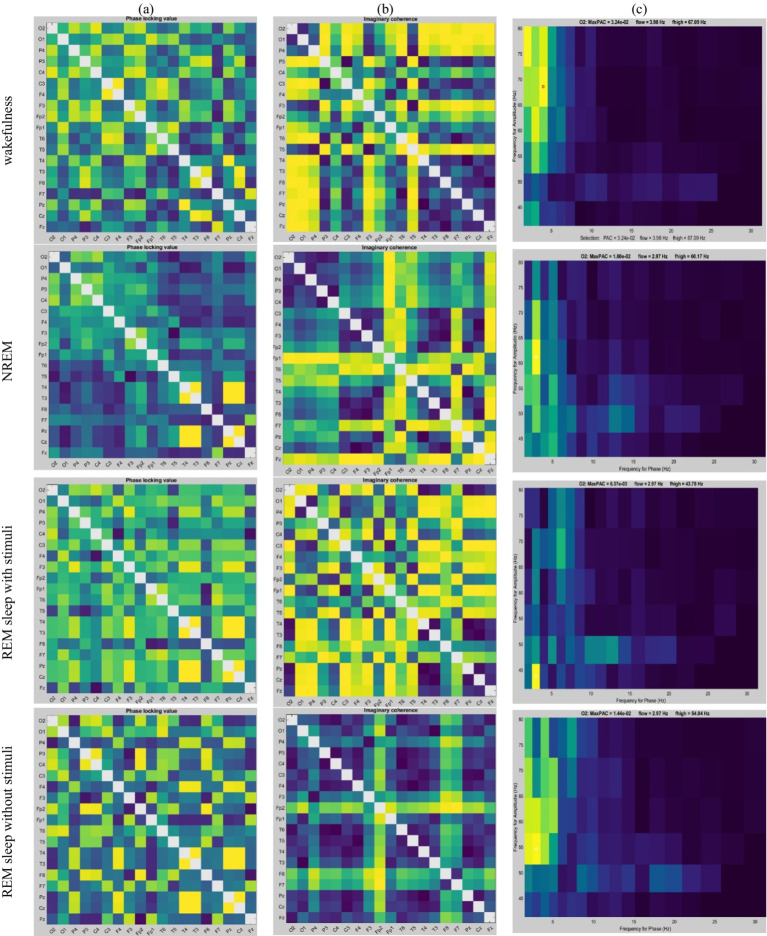
An example of changes in **(a)** PLV, **(b)** Coherence, and **(c)** PAC values depend on each state: wakefulness, NREM, REM sleep with stimuli, and REM sleep without stimuli, respectively for 2 s of EEG signals.

Sample adjacency matrices based on PLV, coherence, and PAC are displayed in Figure 5. These features are subsequently analyzed in the feature selection step. Significant features are then converted into a graph representation and used to train the proposed GICA model.

- RQA: Recurrence refers to the trajectory returning to its previous state in the phase space. The phase space is typically constructed from a time-series signal using a time-embedding method. To visualize the amount of recurrence in a multi-dimensional dynamic system, a recurrent plot (RP) is used. In Equation 3, *RQA* is calculated for each sample, *i*, *j* of the time series *x*, under the predefined threshold distance ε (Kang et al., 2021):

(3) $$RQA_{i,j}=\Theta \left(\varepsilon -\left\|x_{i}-x_{j}\right\|\right),\;\;\;i,j=1,2,\ldots ,N,$$

**Figure 6:**
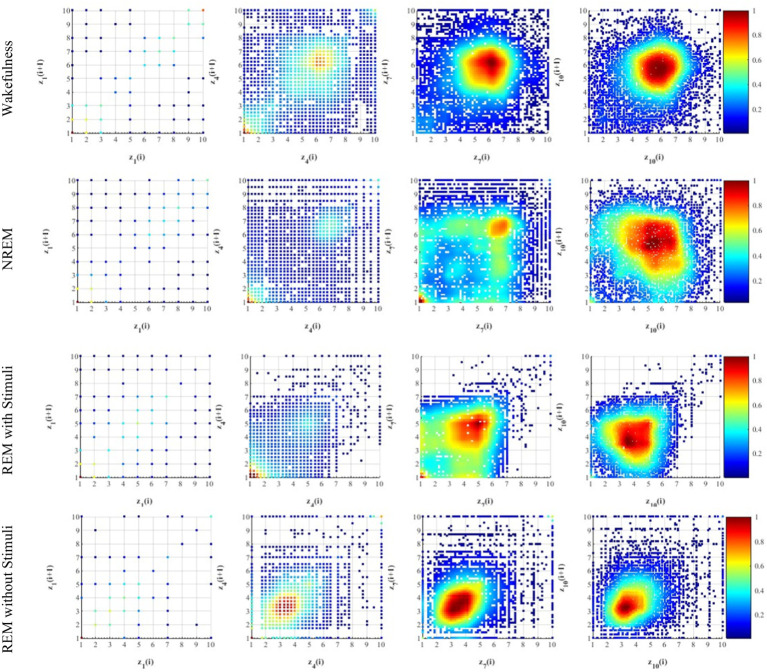
Example of recurrence plots (RP) on four different thresholds extracted from EEG signals during the four different sleep states. Here, the visualization of RP indicates a recurrence ( R(i,j)=1 ) at coordinates (*i*, *j*) with time on both the *x*- and *y*-axes using colored dots. *z*_1_ is recurrence pattern corresponding to short-term phase synchronization, *z*_4_ indicates recurrence pattern representing intermediate-term phase dynamics, *z*_7_ represents recurrence patterns associated with long-term phase stability, and *z*_10_ is characterized by highly dynamic and unstable brain states. Increasing recurrence density from *z*_1_ to *z*_10_ suggests more prolonged sleep stability.

The RQA method was applied to analyze the recurrence of phase in EEG time series. The instantaneous phase was computed using Hilbert transform, and recurrence plots were constructed by comparing phase values at different time points. Phase errors were quantified using a continuous measure of Euclidean distance, as described in Equation 3. This approach avoids the need for binning and provides a precise quantification of recurrence patterns. Figure 6 illustrates the recurrence patterns identified in our analysis, with labels *z*_1_, *z*_4_, and *z*_7_ corresponding to short-term, intermediate-term, and long-term phase dynamics, respectively.

Where Θ(.) , ‖.‖ , and *N* are the Heaviside function, the maximum norm, and the number of samples in the phase space trajectory, respectively. In other words, RP is a two-dimensional representation characterizing the dynamic features of nonlinear systems and complex time series by which the phase space trajectory returns roughly to its previous states (Zheng et al., 2024). Phase space vectors are reconstructed from a given time series (i.e., $x_{1},x_{2},x_{3},\ldots ,x=n$ ) using time-delay embedding methods, such as the Takens time-delay embedding technique (Takens, 2006). Phase space vector is obtained as $X_{i}=\left(x_{i},x_{i}+\tau ,\ldots ,x_{i}+\left(m-1\right)\times \tau \right)$ which parameters *m* and *τ* should be set for phase space reconstruction. Then, the distance in the phase space between $x_{i}$ and $x_{j}$ falls within the *ε*, two samples are considered to be recurrences, indicated as $RQA_{i,j}$ . Recurrence Rate (RR) can be obtained to quantify the RP which measures the percentage of recurrence points in the RP which is calculated as Equation 4 (Wallot, 2011):


(4) $$RR=\frac{1}{N^{2}}\sum_{i,j=1}^{N}RQA_{i,j}.$$

### 2.6 Feature selection

To assess the normality of feature vectors, the Kolmogorov–Smirnov (KS) test is employed as an initial data analysis (Weiss, 1984). The probability values below 0.05 indicate that the data exhibits non-normal distributions. In situations where the data does not follow a normal distribution, the Mann–Whitney U (Wilcoxon rank sum) test is chosen to compare differences between different states of recording EEGs. *p*-values < 0.05 indicate greater significance in terms of significant disparities in medians among the various tasks.

In this research, EEG features were extracted from five frequency bands: delta (0.4–4 Hz), theta (4–8 Hz), alpha (8–12 Hz), beta (12–30 Hz), and gamma (30–70 Hz). Four functional connectivity features were computed for each band: PLV, Coherence, PAC, and RQA. For each 19-channel EEG window, the pairwise connectivity metrics (PLV, Coherence, PAC) resulted in matrices of dimensions 19 × 19 or 8 × 8, while RQA was computed as a 1 × 10 vector, representing recurrence measures extracted from specific EEG microstates. PAC was computed between eight frequency pairs (i.e., *δ*–*θ*, *δ*–*α*, *δ*–*β*, *δ*–*γ*, *θ*–*α*, *θ*–*β*, *θ*–*γ*, and *α*–*γ*) in predefined cortical regions of interest, forming an 8 × 8 connectivity matrix. RR values were extracted from EEG RQA applied to 10 predefined EEG microstate features, summarizing recurrence properties of the EEG time series. To select significant features, statistical analysis (Mann–Whitney U, *p*-values < 0.05) was conducted across groups to identify the most discriminative features. For each matrix-based feature (PLV, Coherence, PAC), the upper triangular portion (excluding the diagonal) was extracted and flattened into a 1D array. This ensured that redundant or symmetrical values were not included in the analysis. This resulted in 171 unique pairwise values for PLV, 171 for Coherence, and 28 for PAC per frequency band. The flattened 1D array features were considered to analyze by statistical test. Only significant features were retained for further classification. This structure feature extraction approach ensures that only informative and statistically relevant connectivity and recurrence measures are used for subsequent analysis.

Principle Component Analysis (PCA) was selected to handle feature correlations and reduce the dimensionality of the dataset (Rahman et al., 2020). PCA is well-suited for this task as it transforms correlated features into a set of orthogonal components, thereby retaining the most relevant information while addressing multicollinearity. Prior to applying PCA, all features were standardized to have zero mean and unit variance. This step is essential because PCA is sensitive to the scale of input features and standardization ensures that all features contribute equally to the principal components. Then, PCA was applied to the standardized data, and components were selected based on the cumulative explained variance. We retained the components that explained 95% of the total variance. This threshold was chosen to balance dimensionality reduction with the preservation of sufficient variance for accurate modeling. This reduction minimized the risk of overfitting, simplified the model, and improved the addressing of potential multicollinearity issues among the original features. The transformed principle components were then used in the detection model, enhancing performance by providing uncorrelated inputs.

In this study, the flattened features underwent statistical testing using the Mann–Whitney U test, and only significant feature dimensions were retained for further processing. Subsequently, PCA was applied to the original shapes of the selected feature matrices to reduce dimensionality while preserving 95% of variance. The high-dimensional feature space (originally 418 features) was transformed into a 40-dimensional representation, ensuring that the most relevant connectivity patterns were retained while minimizing redundancy.

### 2.7 Graph generator

To prepare the distinct information for the GICA classifier, the significant features must converted into graph representation (Raeisi et al., 2022). After extracting the initial feature set (PLV, Coherence, and PAC) from the EEG data, we first converted the flattened feature matrices into a 1D array, which was used solely for statistical testing to identify the most discriminative features. After identifying these significant features, we retained their original 2D structure. PCA was then applied to the 418-dimensional feature space, reducing it to 40 principal components that preserved 95% of the variance. The following outlines the procedure for extracting graphs from the output of the feature selection step.

**Figure 7:**
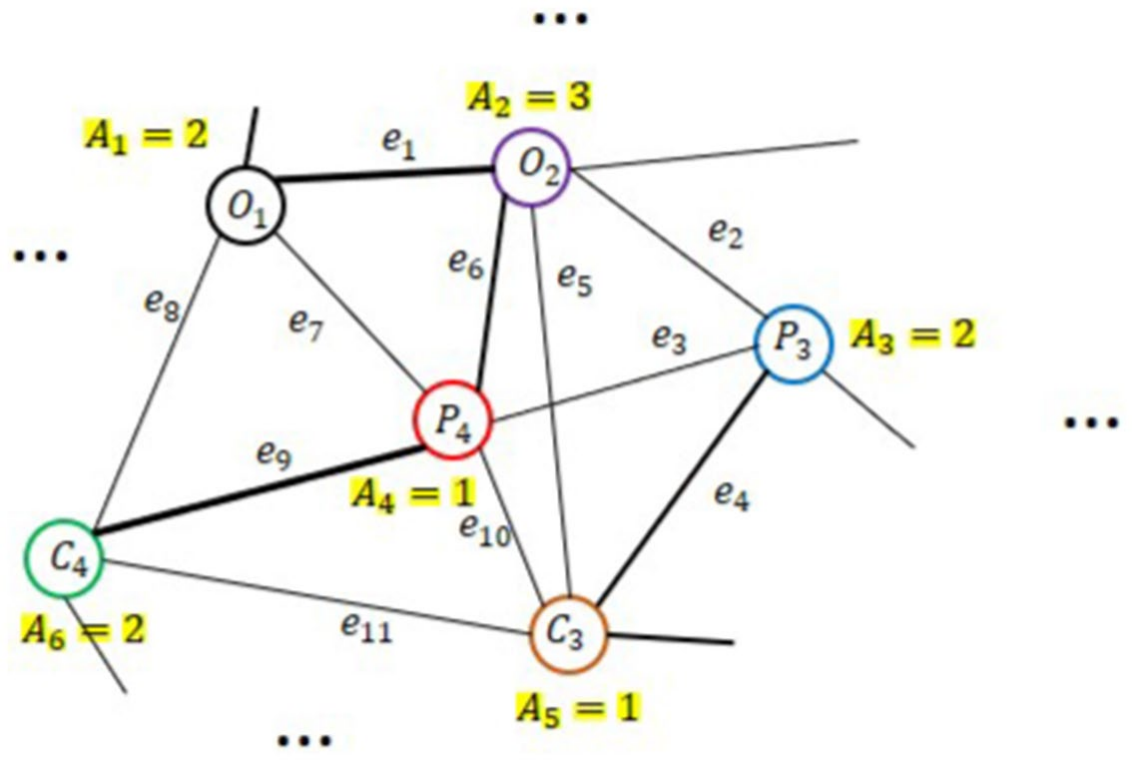
An example of a weighted undirected graph with *V* = 6 nodes (i.e., EEG channels), weighted values *A_i_*, and *e_i_* edges.

Each graph is composed of three elements namely, nodes, connections among them as edges, and sets of features at nodes as graph signals. An undirected weighted graph is symbolized as G={V,E,A} representing the correlations between nodes. Figure 7 shows an example of an undirected graph, where each vertex has a weight $A_{i}$ . Here, $V=\left\{v_{1},v_{2},\ldots ,v_{p}\right\}$ denotes the ensemble of *p* nodes, $e_{i}\in E$ is represented the set of *e* edges, and $A\in R^{p\times p}$ is the weighted adjacency matrix and indicates the linkages between any pair of nodes. The symmetric normalized graph Laplacian $L_{norm}$ is defined as (Xue et al., 2024):


(5) $$L_{norm}=I-D^{-1/2}AD^{-1/2},$$

where **I** is the identity matrix and $D_{i,j}=\sum_{j}A_{i,j}$ is the degree matrix of graph *G*. To illustrate the degree matrix of the graph, we consider the scale of graph weights, regardless of correlation direction. Therefore, we utilize the absolute value of the Pearson correlation coefficient (PCC) matrix. The PCC matrix represents each node in the graph, and the edge weights are determined by the correlations observed among the time-series signals in Equation 6 (Xue et al., 2024):


(6) $$w_{i,j}=\left|\frac{\sum_{t=1}^{T}\left(x_{i}^{t}-{\bar{x}}_{i}\right)\left(x_{j}^{t}-{\bar{x}}_{j}\right)}{\sqrt{\sum_{t=1}^{T}{\left(x_{i}^{t}-{\bar{x}}_{i}\right)}^{2}}\sqrt{\sum_{t=1}^{T}{\left(x_{j}^{t}-{\bar{x}}_{j}\right)}^{2}}}\right|.$$

Here, $x_{i}$ and $x_{j}$ are the signal vectors from nodes $v_{i}$ and $v_{j}$ . The parameter *T* and $\bar{x}$ represent the total number of samples and the arithmetic mean of signal vector from related node. $w_{i,j}\in \left[0,1\right]$ can quantify the relationship between two channels and evaluate the strength of their correlation. A higher $w_{i,j}$ value indicates a stronger correlation between the channels.

The weighted adjacency matrix, $A_{i,j}=w_{i,j}$ , is used to construct the symmetric graph, $L_{norm}$ , as defined in Equation 5. It is important to highlight that the graph representation was generated after applying PCA (Section 2.6) on the extracted significant features, reducing them to a 40-dimensional space. This approach ensures that the graphical representation effectively captures both the topological relationships between features and the informative feature embeddings over time, preserving the most discriminative characteristics for classification. However, when one type of functional connectivity was considered for classification, PCA was not applied. PCA was only applied to the concatenated version of the feature set to reduce dimensionality.

The final version of the GICA model employs a graph representation of features that have been reduced to 40 dimensions through PCA for the classification of sleep states. Prior to finalizing the GICA model, we assessed the model’s performance using each functional connectivity feature individually. The graph, **L**_norm_, was generated for each functional connectivity feature without incorporating PCA, while, **L**_norm_ was also computed from the 40-dimensional PCA-reduced feature set based on the concatenated version of features. The high-dimensional feature space (i.e., PLV + Coherence + PAC measures) was reduced to 40 dimensions using PCA. Each of these 40 dimensions corresponds to a distinct node in the graph, with node attributes reflecting the principal components derived from the original feature set. The graph generator was applied to these 40D PCA-reduced features instead of the original functional matrices of “19×19 + 19×19 + 8×8.” Overall, in assessing classifier performance using a single feature type, we employed graph representation without PCA. Therefore, the GICA model receives a graphical representation of the data in two different input sizes; individual functional connectivity feature dimension or a 40-dimensional PCA-reduced feature set as input.

### 2.8 Classification

A convolutional neural network is a specific type of neural network designed to learn informative features through local receptive fields (Li et al., 2021). It consists of various layers stacked together in a deep architecture, including the input layer, convolutional and pooling layers (which can be combined in different ways), fully connected hidden layers, and an output (loss) layer (Bologna, 2019; Zhao et al., 2024). The strength of CNNs lies in their ability to extract information or features from a given dataset using kernel filters.

On the other hand, autoencoders (AEs) are neural networks that, like many other neural network architectures, utilize the backpropagation algorithm for latent feature learning (Pratella et al., 2021). They are mainly used for unsupervised learning tasks, meaning they do not require labeled data during training. In contrast, CNNs and RNNs are often used for supervised or semi-supervised tasks that rely on labeled data. This makes AEs suitable for situations where labeled data is scarce or expensive to obtain. AEs are designed to automatically learn latent features in an unsupervised manner, typically for tasks such as data compression or dimensionality reduction. However, other neural network architectures, like CNNs, are also capable of learning features from raw data, particularly in supervised contexts like image classification. This encourages the AEs to capture the crucial characteristics of the input data in its encoding, thereby learning a meaningful representation of the data in the latent code (Zhang et al., 2022). AEs also provide various benefits, including dimensionality reduction across different machine learning and data analysis applications, particularly for complex high-dimensional data. They are equally valuable in data compression, encoding information efficiently for storage or transmission, making them particularly useful in resource-constrained applications.

**Figure 8:**
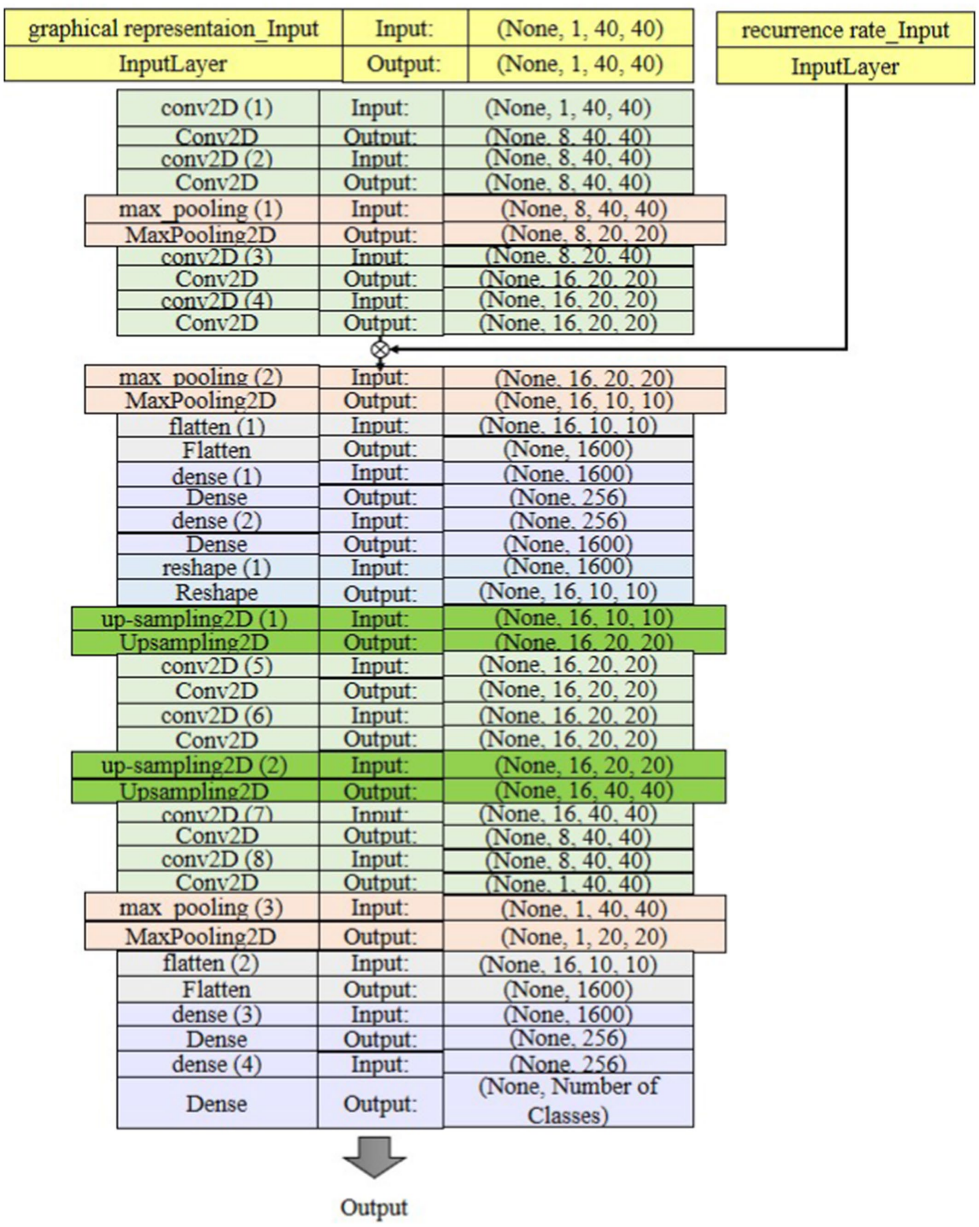
The suggested classification architecture in the proposed GICA model.

The deep learning model proposed for classifying sleep states is depicted in Figure 8. In this model, the pooling layer is incorporated to reduce the output dimension from the convolutional layer, thereby mitigating computational burden and preventing overfitting. Specifically, the max-pooling operation is used to identify the maximum value within each feature map. SoftMax is used to predict which class the extracted feature belongs to. Input data was split to 70% for training, 15% for validation, and the rest for test set. It also utilized 10-fold cross-validation to evaluate and verify the results of the model. Additionally, it reduces the risk of model overfitting and improves the generalization ability of the model. The input data of the first layer is organized in three dimensions: depth, height, and width of the image or r×m×m , where the height (m=40) is equal to the width in one channel data (e.g., RGB image). This represents the features, in this case, the images, in the dataset. “None” represents the batch size, which is *None* here because it can vary depending on how many samples we have in the last batch. In the first Conv2D block, there are two 2-D convolutional layers followed by a max-pooling 2-D layer. It selects the pixel with the highest value for the output array as the filter slides through the input. The RR values are multiplied by each column of data at the output of this layer to emphasize the importance of the temporal components. Another set of Con2D layers outputs a feature map with 16 filters, extracting more abstract patterns in the data. This same pattern repeats a few times until we reach the flattened layer, which connects the output of the previous layer to the Dense layer. 256 neurons in the first dense layer, which processes the flattened data. Another Dense layer with 256 units to refine the learned features. The reshape layer converts the 1-D vector back into a 3-D shape for up-sampling. These layers increase the spatial dimensions of the feature maps. This is useful for reconstructing high-resolution outputs in AEs. After up-sampling, there are additional Conv2D layers to refine the feature maps after resizing them. This helps to generate more accurate reconstruction predictions. Additionally, pooling layers down-sample again, refining features for the final output. The last Dense layer likely corresponds to the output layer, with the number of classes as the output dimension. It connects to the number of possible output classes in a classification task. The proposed network first encodes the input through a series of Conv2D, max-pooling, and Dense layers, compressing the spatial and feature information. Then, it expands the features through up-sampling and Conv2D layers to generate the output.

The input to the proposed GICA model is a 40 × 40 × 1,200 tensor, where 40 × 40 represents the graph matrix constructed based on the 40D PCA-reduced features. The 1,200 corresponds to the number of 2-s EEG segments (since we extract features from each 2-s time window). This indicates that the graphical representation is computed between the 40 principal components rather than the original 19 EEG channels. As shown in Figure 8, the architecture consists of an input layer with shape (1, 40, 40) representing grayscale EEG images. It includes two blocks of Conv2D layers, each with 8 filters, a 2 × 2 kernel size, strides = 1, and ReLU activation. After each Conv2D block, a MaxPooling2D layer with 2 × 2 pooling size is applied for dimensionality reduction. Following two series of these blocks, a Flatten layer reshapes the data for Dense layers, including one with 256 neurons and ReLU activation. The final output layer has 4 or 18 neurons with a Soft-max activation for multi-class classification. A dropout rate of 25% was applied to the fully connected layer to prevent overfitting by reducing neuron co-adaptation. The model is trained with the Adam optimizer (learning rate = 0.001) and categorical cross-entropy loss function over 50 epochs with batch size 32. An L2 regularization term (*λ* = 1 × 10^−6^) was introduced to discourage large weight values and improve generalization. The training process was monitored using a validation set, and early stopping was implemented with a patience value of 100 epochs to terminate training when validation performance plateaued. Ten-fold cross-validation was applied to evaluate model performance, with early stopping to prevent overfitting.

## 3 Experiments and evaluations

### 3.1 Experimental setup

In this work, two experiments are conducted to evaluate the performance of the proposed GICA method based on multivariant graphical representation and RR features. The evaluation of the classification procedure using different features extracted by PLV, coherence, PAC, and RR, separately in the first experiment. Here, the EEG signals of 33 subjects during the four functional states were selected to analyze the efficiency of the GICA classifier. Functional connectivity measures and recurrence quantification analysis are obtained on the input EEG signals of non-overlap windows along 250 samples. These features integrate both local and global information through the concatenation of two 19-channel connectivity features (PLV and Coherence), 8-D PAC values in significant frequency bands, and 1-D RR values at the 10th diameter. To produce a square unit with equal dimensions for vertical and horizontal axes, zero padding is applied. The extracted features are given to the GICA classifier in different structures for detecting brain behavior, separately. This is performed to find an appropriate classifier structure with high performance in sleep classification from EEGs.

**Figure 9:**
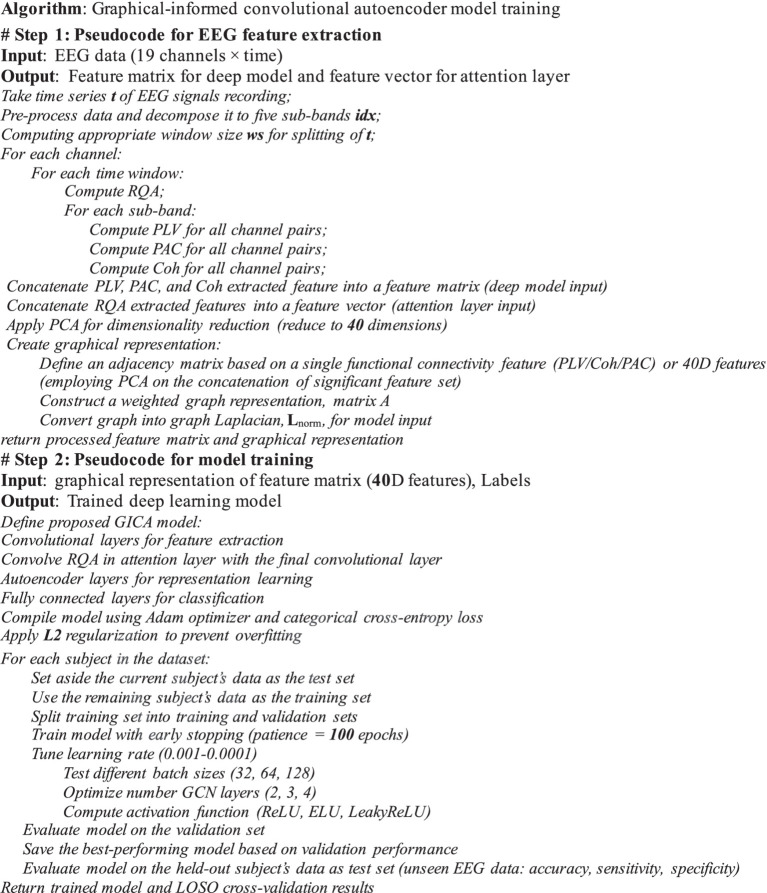
The pseudocode of feature extraction and training phase of the deep learning model.

In the second experiment, we assess the efficiency of the multivariate graph analyzer by emphasizing RR features, such as the attention block in the deep model. In this case, we feed the significant functional connectivity measures extracted from EEG sub-bands to the GICA classifier in its optimal structure. To enhance the reproducibility of our study, we provide a structured pseudocode outlining the feature extraction and deep-learning model training. This pseudocode details the sequence of operations, including EEG pre-processing, feature computation (PLV, PAC, Coh, and RQA), dimensionality reduction via PCA, and construction of the GICA. The complete pseudocode is provided in Figure 9. In Step 1, following pre-processing, the functional connectivity features (i.e., PLV, Coherence, and PAC) and recurrence quantification were extracted across various EEG frequency sub-bands. Statistical test were employed to identify distinct features among these. PCA was applied to the concatenated version of these features; however, PCA was not utilized for assessing classification performance on individual functional connectivity features. Therefore, if the solely feature was considered to feed the GICA model, the graph representation is generated in the same size as the feature. However, if the 40D PCA-reduced features were considered to feed the classifier, the graph **L**_norm_ in Equation 5 was generated with 40D nodes. The proposed GICA model was evaluated using two types of features: individual connectivity feature and a 40D PCA-reduced feature set. In the second step, the deep model was trained and tested using optimized hyperparameters. Finally, the evaluation metrics were computed and reported.

For both experiments, we use 70% of the data (i.e., 24.5 min × 32 subjects) as the training set, while the remaining data is considered as the test set. We employed leave-one-subject-out cross-validation (LOSO) (Roth, 2004), wherein each subject’s data is held out as a test set, while the model is trained on the remaining subjects’ data. This approach minimizes subject-dependent bias by ensuring that no individual data is used for both training and testing simultaneously. To evaluate the performance of our proposed method, we simulate and use the recently developed sleep state detection systems introduced by Mostafaei et al. (2024), Moctezuma et al. (2024), Li et al. (2022), Al-Salman et al. (2023), Eldele et al. (2021), and EEGNet (Lawhern et al., 2018) as baseline systems from the literature to assess the effectiveness of various deep neural architectures. Mostafaei et al. (2024) developed a transformer encoder-decoder model that utilizes an attention block to classify sleep stages based on effective input data patterns. Moctezuma et al. (2024) employed a GRU architecture to capture long-term dependencies in EEG data. Their proposed gating mechanism facilitates the updating and retention of information over time, allowing the GRU layers to enhance the classifier’s ability to detect more complex patterns. However, increase in model capacity can lead to overfitting. Li et al. (2022) designed a multi-layer CNN to extract time and frequency features from EEG spectrograms. Their model culminated in a global average pooling layer followed by two bi-directional long short-term memory layers, which learn the transitional relationships between adjacent sleep stages for classification. In contrast, Al-Salman et al. (2023) distinguished their model from other baselines by employing a machine learning approach rather than deep learning to identify six sleep stages from EEG signals. They utilized discrete wavelet transforms (DWT) to decompose the data into wavelet coefficients and classified the probability distributions of k-means clustered features into sleep stages using a least-square support vector machine (LS-SVM). Eldele et al. (2021) introduced a multi-resolution CNN (MRCNN) with adaptive feature recalibration to extract both low and high-frequency features. They developed multi-head attention as a core component of the attention temporal context encoder to capture long-term dependencies in the input features. EEGNet (Lawhern et al., 2018) is an open-source toolbox based on a CNN architecture for EEG-based BCIs, designed to be trainable with limited data while producing neurophysiologically interpretable features. However, these architectures did not utilize their original feature extraction methods; instead, they were provided with the same graphically informed features, focusing solely on the classification model’s ability to process the structured feature space, rather than the differences in feature extraction. The hyperparameters were either adopted from their original publications.

### 3.2 Evaluation criteria

The efficiency of the proposed GICA algorithm is determined through accuracy (Acc), sensitivity (Sen), and specificity (Spe) (Zhu et al., 2010). Acc indicates the overall correct detection. Sen shows the rate of correctly classified states, while Spe measures the rate of correctly rejected states. Based on the values of true positives (TP), true negatives (TN), false positives (FP), and false negatives (FN), the overall Acc, Sen, and Spe can be defined as follows in Equations 7-9 (Zhu et al., 2010):


(7) $$Acc=\frac{TP+TN}{TP+TN+FP+FN},$$

(8) $$Sen=\frac{TP}{TP+FN},$$

(9) $$Spe=\frac{TN}{TN+FP}.$$

## 4 Results and discussion

Two experiments are conducted to determine the optimal procedure for identifying sleep states using functional connectivity, graph computation, and recurrence quantification analysis of the brain. In the first experiment, a statistical analysis is performed on all individual features to find the significant differences (*p*_value < 0.05) between the various states. The second experiment utilized a graph-informed convolutional autoencoder to evaluate the effectiveness of each multivariate feature set. Additionally, the impact of the different durations of EEG segments is assessed on the performance of the proposed method. In the following analysis, classifier performance was evaluated using a single feature type without the application of PCA for graph representation. However, when a combination of significant features is utilized for classification, PCA is applied to reduce dimensionality before constructing the graph matrix.

### 4.1 Statistical analysis

**Figure 10:**
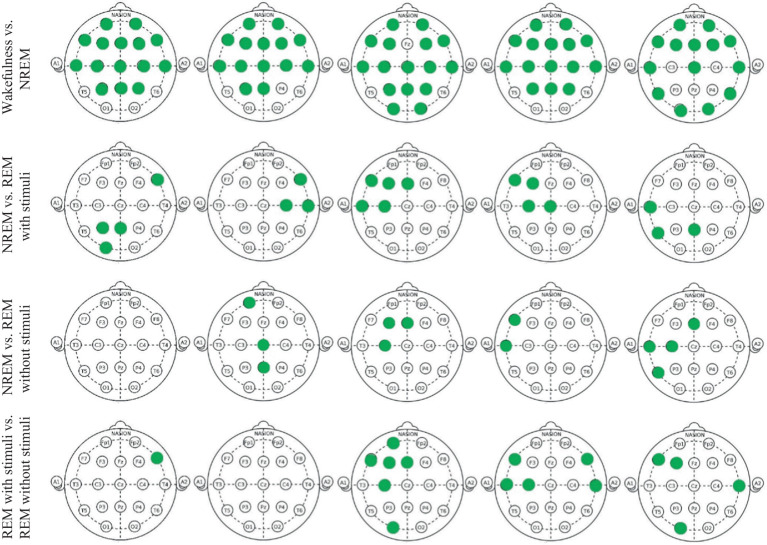
Green points indicate EEG channels with significant differences (*p*_value < 0.05) between two groups in each delta, theta, alpha, beta, and gamma band, respectively.

Figure 10 and Table 1 show the significant *p*_values for extracted PLV and coherence from each sub-band of every single electrode of EEG signals during the wakefulness, NREM sleep, REM sleep with stimuli, and REM sleep without stimuli phases. According to the figure, the most significant effects of PLVs are in the alpha band on the left frontal lobe, F3 and C3 channels between various states of EEG recordings. In addition, *F7* has significant differences in the beta band. It reveals that playing or not playing with auditory stimuli affects the frontal, pre-motor, and auditory areas during sleep. However, other EEG sub-bands such as theta, alpha, and beta have no distinguishable areas except during the comparison of wakefulness and NREM states. The results of Table 1 show that there is a significant coherence between different sub-bands, particularly in the alpha and beta ranges, during sleep with stimuli and other states. Tables 2, 3 depict the disparities between PAC and RR features across EEG sub-bands in the four sleep states. As observed in these tables, the extracted PAC features from the alpha band exhibit significant differences between the REM sleep state and the others. On the other hand, the gamma band shows the most notable distinctions among the various sleep states. It should be noted that the results in Figure 10 demonstrate the significant differences among each electrode; however, the results in Tables 1–3 indicate significant differences across the whole electrode set. The tables present *p*-values computed for all flattened pairwise connections of connectivity measures between various states.

**Table 1:** *p*-values and effect size of Mann–Whitney test for extracted coherence feature from EEGs.

State		Delta	Theta	Alpha	Beta	Gamma
Wakefulness vs. NREM	*p*	0.052	0.300	**0.042** ^*^	**<0.001** ^*^	**0.001** ^*^
	*η* ^2^	<0.001	<0.001	0.11	0.08	0.07
NREM vs. REM with stimuli	*p*	0.410	0.658	**0.037** ^*^	0.322	0.735
	*η* ^2^	<0.001	<0.001	0.11	<0.001	<0.001
NREM vs. REM without stimuli	*p*	**<0.001** ^*^	**<0.001** ^*^	0.665	0.788	0.089
	*η* ^2^	0.16	0.20	<0.001	<0.001	<0.001
REM with stimuli vs. REM without stimuli	*p*	**<0.001** ^*^	0.577	**<0.001** ^*^	**0.003** ^*^	**0.045** ^*^
	*η* ^2^	0.14	<0.001	0.21	0.12	0.09

The symbol * indicates a significant difference (*p*_value < 0.05). Significant values are in [bold].

**Table 2:** *p*-values and effect size of Mann–Whitney test for extracted PAC feature from EEGs.

State		Delta	Theta	Alpha	Beta	Gamma
Wakefulness vs. NREM	*p*	**<0.001** ^*^	0.210	0.512	0.168	**0.021** ^*^
	*η* ^2^	0.25	<0.001	<0.001	<0.001	0.09
NREM vs. REM with stimuli	*p*	0.085	0.492	**0.008** ^*^	0.241	0.735
	*η* ^2^	0.002	<0.001	0.14	<0.001	<0.001
NREM vs. REM without stimuli	*p*	0.071	0.566	**<0.001** ^*^	**0.018** ^*^	0.191
	*η* ^2^	0.01	<0.001	0.21	0.11	<0.001
REM with stimuli vs. REM without stimuli	*p*	0.051	0.094	**<0.001** ^*^	**0.010** ^*^	**0.005** ^*^
	*η* ^2^	<0.001	<0.001	0.16	0.12	0.11

The symbol * indicates a significant difference (*p*_value < 0.05). Significant values are in [bold].

**Table 3:** *p*-values and effect size of Mann–Whitney test for extracted RR feature from EEGs.

State		Delta	Theta	Alpha	Beta	Gamma
Wakefulness vs. NREM	*p*	0.294	0.981	**<0.001** ^*^	0.914	**<0.001** ^*^
	*η* ^2^	<0.001	<0.001	0.22	<0.001	0.18
NREM vs. REM with stimuli	*p*	0.147	0.408	0.068	0.238	0.784
	*η* ^2^	<0.001	<0.001	0.09	<0.001	<0.001
NREM vs. REM without stimuli	*p*	0.306	0.252	0.337	0.058	**0.019** ^*^
	*η* ^2^	<0.001	<0.001	<0.001	0.02	0.10
REM with stimuli vs. REM without stimuli	*p*	**<0.001** ^*^	0.295	**<0.001** ^*^	0.544	**0.016** ^*^
	*η* ^2^	0.24	<0.001	0.12	<0.001	0.10

The symbol * indicates a significant difference (*p*_value < 0.05). Significant values are in [bold].

According to these findings, the significance of EEG features in the alpha and gamma bands aligns with prior sleep research, supporting their biological plausibility. Alpha activity is associated with wakefulness and drowsiness, typically decreasing during REM sleep (Cantero et al., 2002; Cantero and Atienza, 2000). Some studies suggest that alpha intrusions may indicate sleep instability, particularly in individuals with sleep disorders. Meanwhile, gamma oscillations are linked to cognitive processing, memory consolidation, and neural plasticity during REM sleep. Increased gamma activity during REM is thought to reflect heightened cortical processing and dream-related activity (Mishra and Colgin, 2019; Corsi-Cabrera et al., 2014). These findings further validate the use of EEG-based connectivity measures in sleep-stage classification and support the robustness of our approach.

### 4.2 SlS classification with GICA model

**Figure 11:**
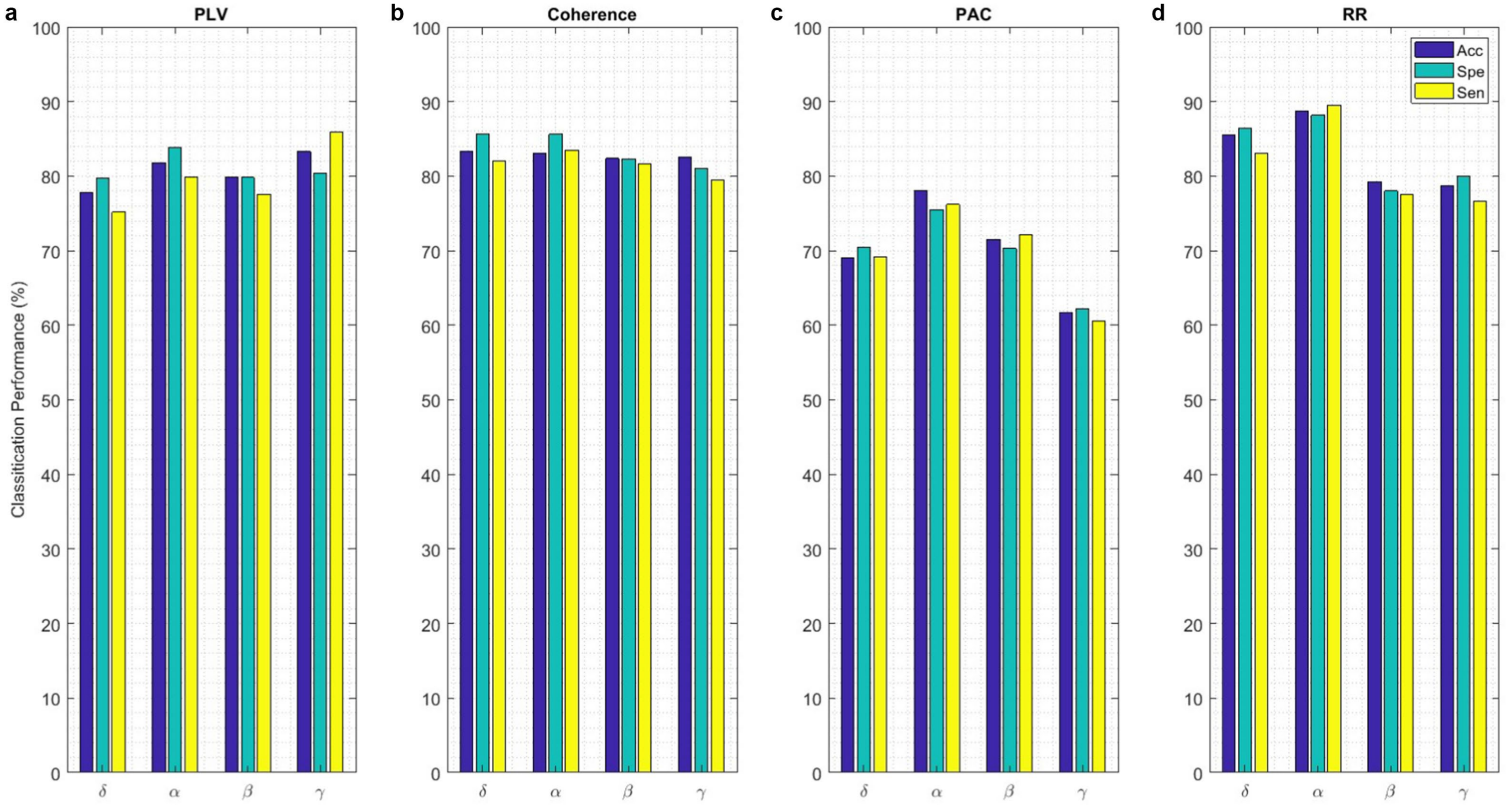
The SlS classification with the proposed GICA model only includes **(a)** PLV, **(b)** Coherence, **(c)** PAC, and **(d)** RR features extracted from EEG signals on different sub-bands. The performance of the SlS classifier is presented based on functional connectivity features, without taking PCA into account. The connectivity measures were utilized for graph generation and subsequently input into the GICA model.

**Figure 12:**
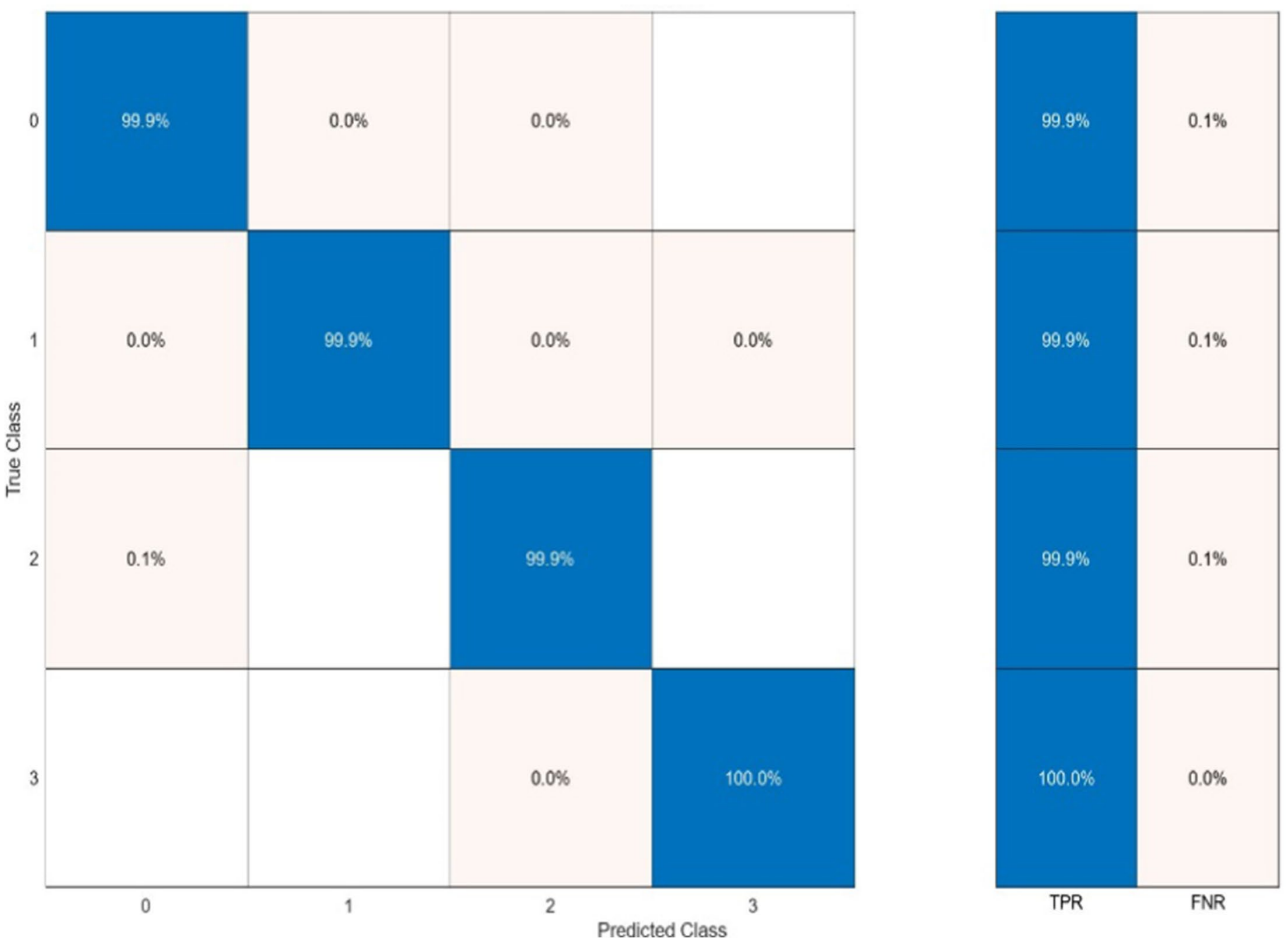
The sleep states classification using the proposed GICA algorithm which categorizes the states as wakefulness (0), NREM (1), REM with stimuli (2), and REM without stimuli (3). The SlS classifier is applied to the graphical representation of a 40-dimensional PCA-reduced significant feature set.

Classification performance acquired from each functional connectivity feature and recurrence quantification analysis are presented in Figure 11. These results include the Acc, Spe, and Sen on EEG signals on extracted features from each 2 s segments. In the sense of classification results for different features, it can be seen that these parameters achieve Acc between 61.7% for classification with PAC features on the gamma band in minimum and 88.8% with RR features on the alpha band in maximum value. To obtain the highest performance of SlS, the best features of EEG sub-bands are selected from the point of view of classification accuracy. These significant features are concatenated, namely, “*PLV_γ_* + *Coh_δ_* + *PAC_α_*” to yield a 57-dimensional feature set. Then, GICA with *RR_α_*-attention block is trained from the concatenated features to classify four sleep states. Figure 12 shows the confusion matrix for SlS classification with the GICA model. Overall, accuracy and a true positive ratio (TPR) of 99.92% are achieved with the proposed GICA model. This indicates that it is excellently suited for detecting sleep states in unseen data. Hence, larger datasets are acquired so the training process can be effective in the initial phase. In conclusion, the analyzed FC and GICA model demonstrate satisfactory performance in the multi-class classification of EEG datasets with relatively unbalanced class proportions.

To ensure the generalizability of the proposed model and minimize the risk of overfitting, LOSO cross-validation was utilized, which ensures that each test fold comprises entirely unseen subjects. This method effectively evaluates the model’s capacity to generalize beyond individual subjects. Additionally, regularization techniques, including dropout layers and batch normalization, were integrated into the model to prevent it from learning spurious correlations in the training data. Furthermore, feature selection through PCA was performed to reduce dimensionality and retain only the most informative components, thereby avoiding overfitting to noise.

### 4.3 BRSl classification with GICA model

**Figure 13:**
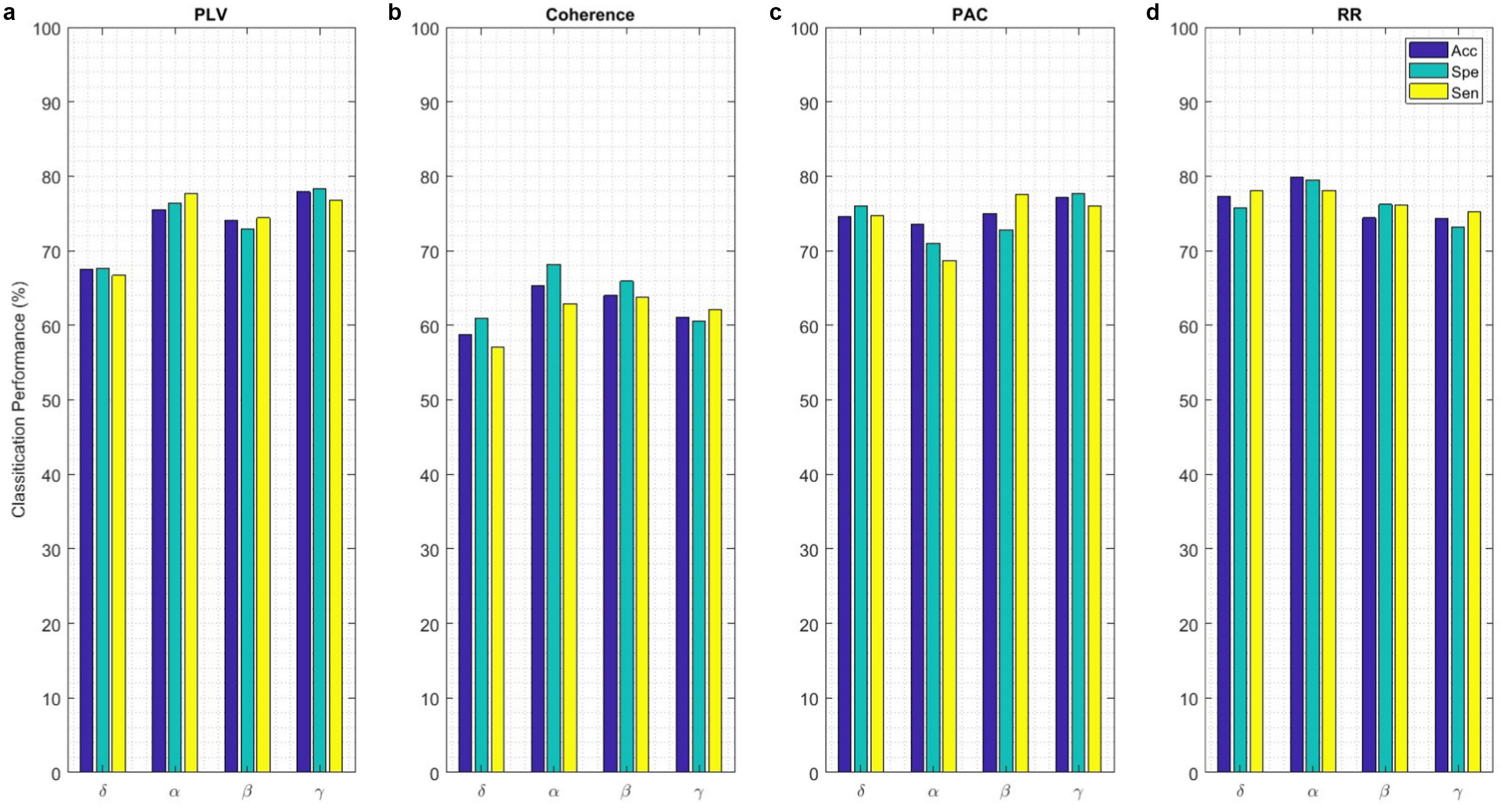
The BRSl classification with the proposed GICA model only includes **(a)** PLV, **(b)** Coherence, **(c)** PAC, and **(d)** RR features extracted from EEG signals on different sub-bands. The performance of the BRSl classifier is presented based on functional connectivity features, without taking PCA into account. The connectivity measures were utilized for graph generation and subsequently input into the GICA model.

**Figure 14:**
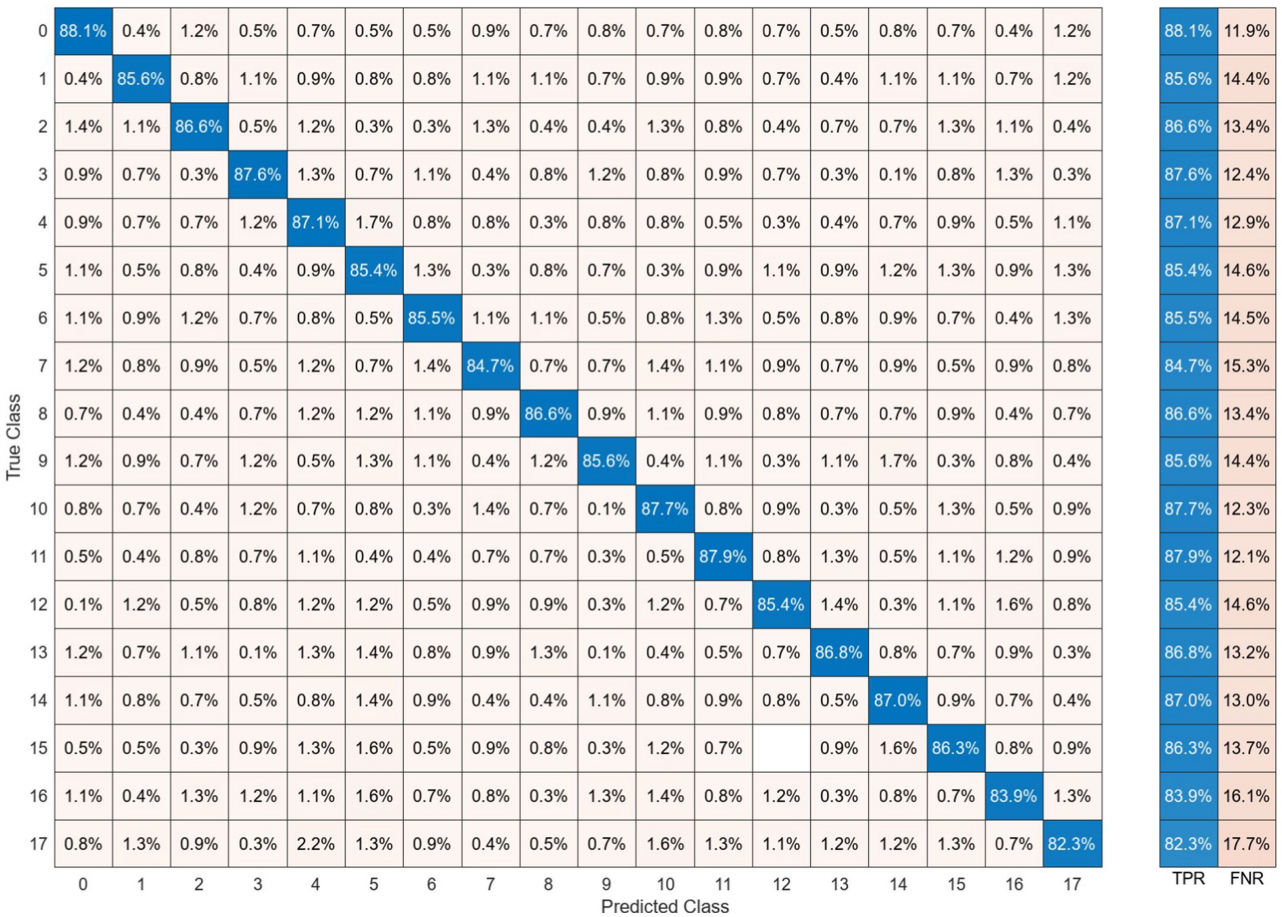
The brain-behavior classification during REM sleep and presenting 18 stimuli using the proposed GICA algorithm on the features of “*PLV_γ_* + *Coh_α_* + *PAC_γ_*.” The BRSl classifier is applied to the graphical representation of a 40-dimensional PCA-reduced significant feature set.

The performance of classifying brain responses to auditory stimuli during REM sleep using different functional connectivity features and recurrence quantification analysis is illustrated in Figure 13. The results of the Acc, Spe, and Sen evaluation metrics on EEG signals are extracted from 2 s segments of each feature. It can be observed that these parameters achieve an accuracy ranging from 58.8 to 79.8% for different features. According to the results presented in this figure, the performances of different features close to each other. This shows the discriminatory efficiency of functional connectivity features obtained by PLV, Coh, PAC, and RR from each EEG sub-band. To obtain the highest performance of BRSl, the best features of EEG sub-bands are selected from the point of view of classification accuracy. These significant features including 19 × (19 − 1)/2 = 171 elements of PLV, 19 × (19 − 1)/2 = 171 elements of Coh, and 8 × 19/2 = 76 elements for PAC are concatenated (total dimension = 418), namely, “*PLV_γ_* + *Coh_α_* + *PAC_γ_*” to yield a 40-dimensional feature space after applying PCA. Then, GICA with *RR_α_*-attention block is trained from the concatenated features (i.e., (32, 40): 32 subjects and 40-dimensional feature vector) to classify brain responses to 18 auditory stimuli during REM sleep. Figure 14 displays the confusion matrix for BRSl classification with the GICA model. Overall, accuracy and a TPR of 86.1% are achieved with the proposed GICA model. This indicates that the brain responses in unseen data are within the acceptable range for detection.

**Figure 15:**
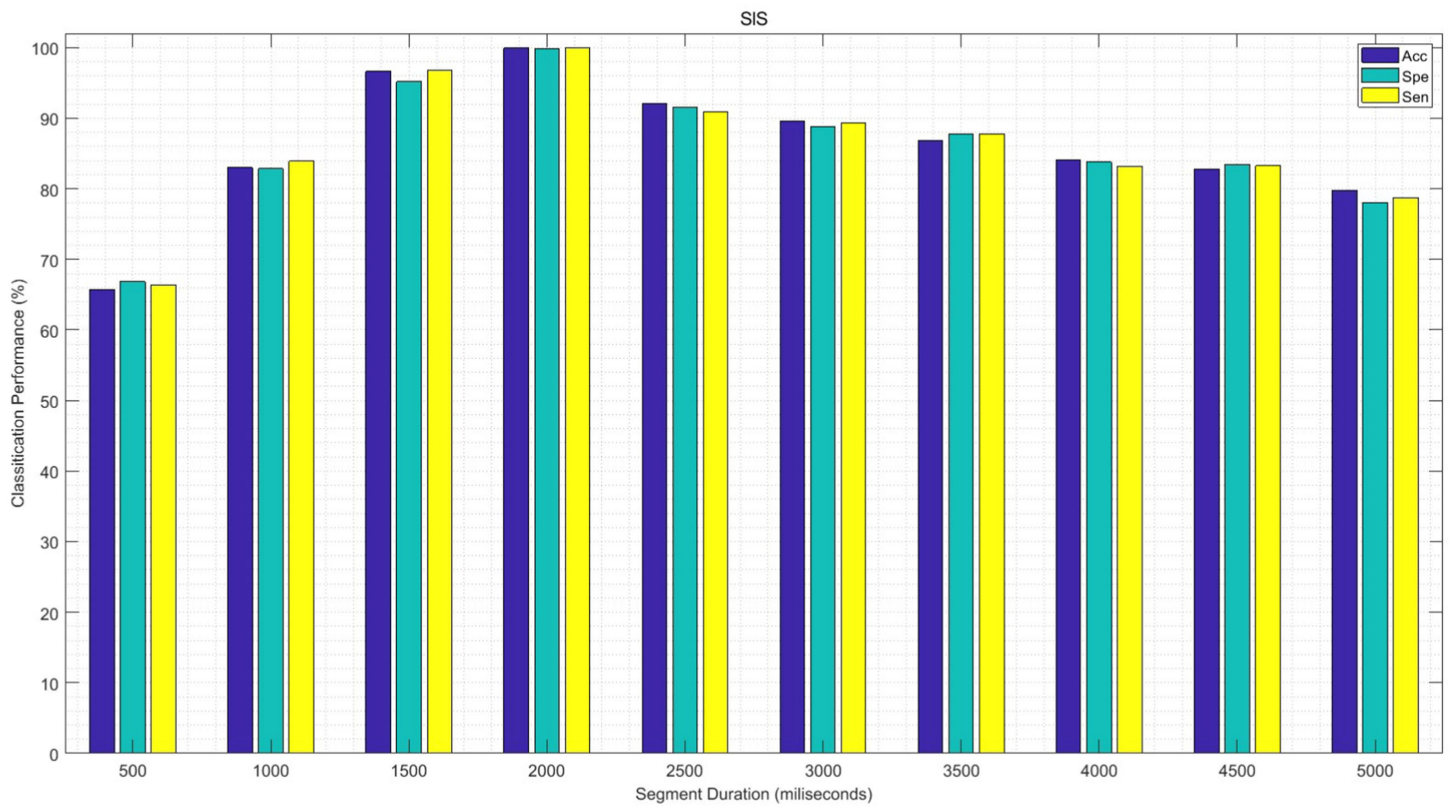
The performance of the SlS classifier is presented based on a significant functional connectivity feature set, including “*PLV_γ_* + *Coh_δ_* + *PAC_α_*.” Following PCA, the 40-dimensional feature set was utilized for graph generation and subsequently input into the GICA model for different EEG processing segments.

In the following experiment, the performance of the proposed GICA model is examined for different segments of EEG signals in sleep state classification. To this aim, first, the functional connectivity features of EEG data are extracted from each sub-band for different durations of EEG segments from 500 ms to 5,000 ms. The significant features are then fed into the GICA classifier. The average of the SlS performances is illustrated in Figure 15 for 100 epochs. It can be observed that the classification performance of the proposed algorithm decreases significantly as the duration of the EEG segment increases for segment durations above 2 s. Additionally, this figure shows that the measures of Acc, Spe, and Sen are increased as the data length is shortened, specifically, in the length of 1,500–2,500 ms. Moreover, the Spe and Sen values lie in acceptable ranges for all EEG segments. These achievements could be considered in real-time and automatic applications to develop new therapeutic strategies for sleep-related disorders.

The impact of each feature type on classification performance has evaluated in ablation study that involved systematically removing one feature type at a time. The results are summarized in Table 4. The removal of PAC led to the most significant drop in accuracy, highlighting its essential role in differentiating sleep states. The model achieved the highest performance when all features were combined, understanding the complementary contributions of PLV, Coh and PAC.

**Table 4:** The ablation study on the feature set with proposed deep learning model in SlS and BRSl classification on α band.

Model Structure and Input Features	SlS classification (%)			BRSl classification (%)		
	Acc	Spe	Sen	Acc	Spe	Sen
Proposed GICA model with PLV + PAC + Coh	**96.50**	95.56	97.28	**86.10**	86.55	84.72
Proposed GICA model with PLV + PAC	96.12	95.50	94.22	85.88	85.45	85.30
Proposed GICA model with PLV	92.83	92.46	92.55	85.25	86.60	83.35
Proposed GICA model with PAC + Coh	92.46	91.13	91.22	84.40	85.90	81.23
Proposed GICA model with PAC	93.80	91.73	93.45	84.86	83.21	85.57
Proposed GICA model with Coh + PLV	91.97	89.85	89.51	82.95	81.14	82.10
Proposed GICA model with Coh	91.95	93.33	92.58	82.97	81.59	82.96

The graph input was generated based on functional connectivity measures without the PCA. Maximum value of Acc are in [bold].

To assess the impact of different model components, we performed an ablation study by evaluating multiple deep learning architectures with varying configurations. Table 5 summarizes the results, showing that the proposed GICA model achieves the highest accuracy while maintaining generalization. Removing dropout and regularization led to overfitting whereas reducing network depth resulted in decreased accuracy. These findings highlight the significance of autoencoder-based extraction and regularization techniques in optimizing model performance. The ablation study confirms that adding graphical input, autoencoder layers, and attention layer improves classification accuracy, likely due to better feature extraction. The removal of dropout and regularization significantly impacted model generalization, leading to overfitting. Similarly, reducing the network depth resulted in lower accuracy, indicating the importance of deeper representation for sleep EEG classification.

**Table 5:** The ablation study and a detailed overview of the different deep learning architecture and their classification results in SlS and BRSl categories on the graphical representation of the 40D PCA-reduced feature set extracted from *α* band.

Model Structure	SlS classification (%)			BRSl classification (%)		
	Acc	Spe	Sen	Acc	Spe	Sen
CNN (4 layers)	89.83	90.25	87.50	77.51	77.20	78.16
CNN (4 layers) + Autoencoder (4 layers)	93.33	91.94	93.82	80.28	80.25	79.56
CNN + Autoencoder + Attention	95.24	96.45	94.10	82.02	81.64	83.05
Proposed GICA	**96.50**	95.56	97.28	**86.10**	86.55	84.72
Proposed GICA without dropout and regularization	98.50	99.98	98.11	85.09	83.31	85.46

Maximum value of Acc are in [bold].

To assess the computational feasibility of our model, we analyzed its processing requirements. The proposed model consists of 67,110,941 trainable parameters and requires 26,831 min for training. The inference time per sample is 36 s, making it suitable for offline analysis. However, real-time applications may require additional optimization techniques, such as model pruning, quantization, or deployment on specialized hardware. All computational time measurements were conducted on a Windows 11-based system equipped with an Intel Core i7 processor and an NVIDIA GTX 1660 GPU.

**Figure 16:**
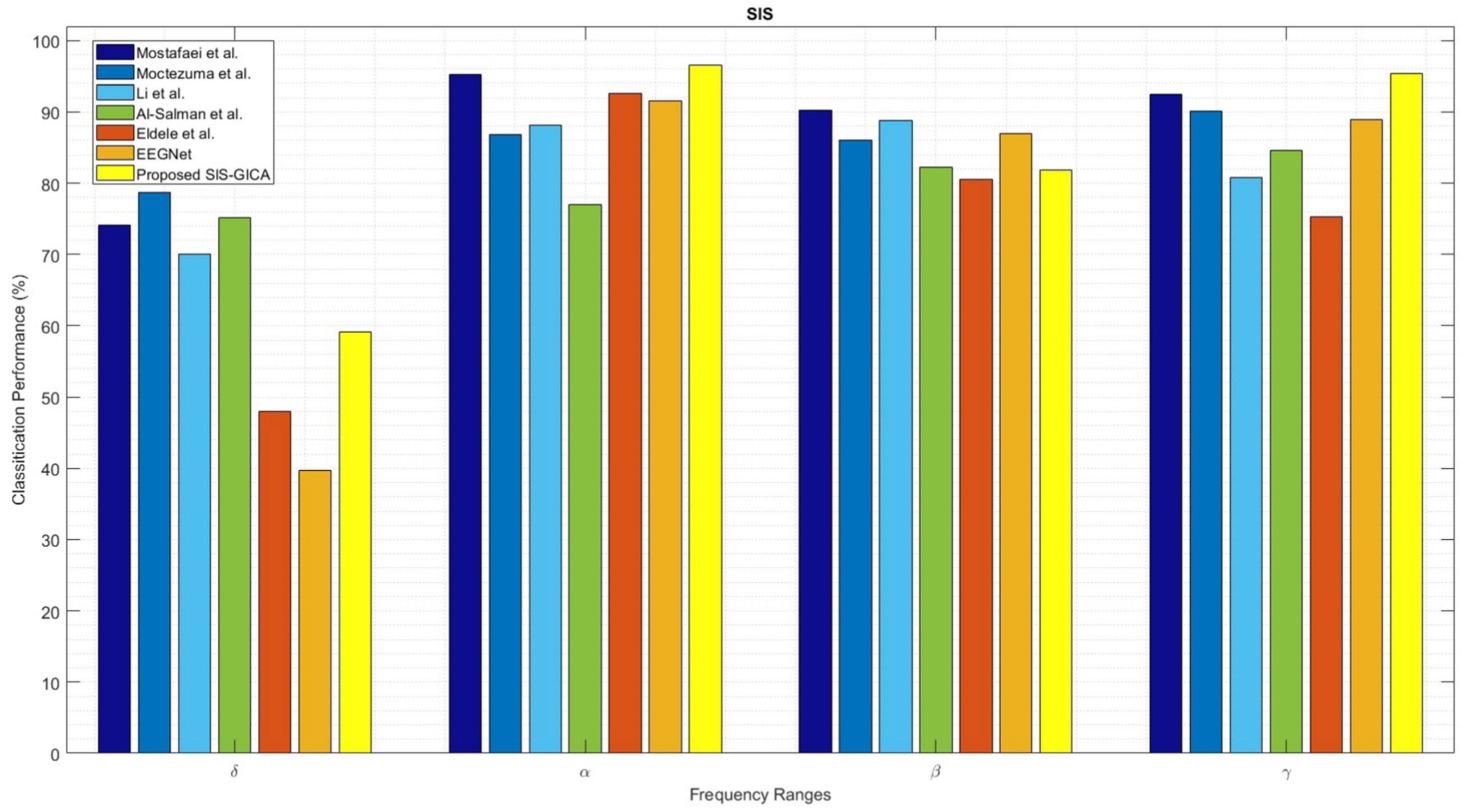
Comparison of the proposed SlS-GICA model on the features of “*PLV_γ_* + *Coh_δ_* + *PAC_α_*” in sleep classification with other baselines including Mostafaei et al. (2024), Moctezuma et al. (2024), Li et al. (2022), Al-Salman et al. (2023), Eldele et al. (2021), and EEGNet (Lawhern et al., 2018). The SlS classifier is applied to the graphical representation of a 40-dimensional PCA-reduced significant feature set (i.e., “*PLV_γ_* + *Coh_δ_* + *PAC_α_*”).

Figure 16 compares the performance of the proposed SlS-GICA model using significant features (PLV, Coh, and PAC) to the baseline systems in terms of accuracy, sensitivity, and specificity measures. The introduced SlS algorithm outperforms the baseline systems including Mostafaei et al. (2024), Moctezuma et al. (2024), Li et al. (2022), Al-Salman et al. (2023), and Eldele et al. (2021) based on the accuracy criteria. In this work, the methods from baselines were reimplemented based on the descriptions provided in their respective papers. The reimplementation followed the original algorithms as closely as possible to ensure a fair comparison. Minor adjustments were made only where necessary to ensure compatibility with the experimental framework used in this study, which was detailed in the material and methods section. The results in Table 6 show that the accuracy of the SlS classification systems generally increases with the frequency ranges. The highest performance (96.50%) is achieved by the functional connectivity extracted from the alpha band with the proposed SlS-GICA classifier. Mostafaei et al. (2024) achieved accuracies of 74.16, 95.25, 90.25, and 92.50% for the *δ*, *α*, *β*, and *γ* frequency bands, respectively.

**Table 6:** Comparison of the proposed GICA model in sleep classification with other baselines including Mostafaei et al. (2024), Moctezuma et al. (2024), Li et al. (2022), Al-Salman et al. (2023), Eldele et al. (2021), and EEGNet (Lawhern et al., 2018).

Models	Metrics	*δ*	*α*	*β*	*γ*
Mostafaei et al. (2024)	*Acc*	74.16	95.24	**90.25**	92.50
	*Sen*	75.89	95.78	89.44	93.42
	*Spe*	73.14	92.57	89.08	94.56
Moctezuma et al. (2024)	*Acc*	**78.75**	89.80	86.10	90.15
	*Sen*	76.52	91.40	87.78	91.25
	*Spe*	74.06	90.71	88.79	92.31
Li et al. (2022)	*Acc*	69.97	86.20	88.85	80.85
	*Sen*	65.20	88.18	86.60	77.36
	*Spe*	69.10	87.09	88.43	79.90
Al-Salman et al. (2023)	*Acc*	75.15	77.05	82.20	84.65
	*Sen*	75.11	76.75	82.50	81.98
	*Spe*	76.08	78.89	81.27	83.50
Eldele et al. (2021)	*Acc*	48.00	92.61	80.60	75.35
	*Sen*	47.10	91.79	81.40	73.30
	*Spe*	46.57	93.50	81.64	72.70
EEGNet (Lawhern et al., 2018)	*Acc*	39.66	91.52	86.93	88.95
	*Sen*	40.57	92.75	85.68	88.50
	*Spe*	38.05	92.97	87.33	87.24
Proposed SlS-GICA classifier	*Acc*	59.11	**96.50**	81.80	**95.33**
	*Sen*	59.48	95.56	80.02	95.70
	*Spe*	60.75	97.28	81.31	94.37

Maximum value of Acc are in [bold].

The SlS detection model of Moctezuma et al. (2024) achieved accuracies of 78.75, 89.80, 86.10, and 90.15% for the FC features extracted from the *δ*, *α*, *β*, and *γ* bands, respectively. Li et al. (2022) obtained accuracies of 69.97, 86.20, 88.85, and 80.85% for the *δ*, *α*, *β*, and *γ* EEG sub-bands, respectively. Al-Salman et al. (2023) model achieved accuracies of 75.75, 77.05, 82.20, and 84.65% for *δ*, *α*, *β*, and *γ* bands, respectively. Eldele et al. (2021) achieved accuracies of 48.00, 92.61, 80.60, and 75.35% for the FC features extracted from the *δ*, *α*, *β*, and *γ* bands, respectively. However, the proposed SlS-GICA model achieved accuracies of 59.11, 96.50, 81.80, and 95.33% for the extracted features from the *δ*, *α*, *β*, and *γ* EEG sub-bands, respectively. The highest accuracy is observed for the proposed SlS-GICA model in the alpha and gamma bands, with accuracies of 96.50 and 95.33%, respectively. Additionally, Mostafaei et al. (2024) achieved an accuracy of 90.25% for the extracted FC features from the beta band. By using a consistent feature input across models, this comparison isolated the influence of network architecture on classification performance, ensuring a fair assessment of our proposed approach.

The exploratory analysis yielded significant results for the functional connectivity and recurrence analysis of EEG sub-bands. Also, the length of EEG processing has a stronger impact on extracting differences in brain function during the seconds. In addition, extracting RR from EEGs and applying it as an attention layer in the GICA model, emphasizes the dynamic behavior of the brain performance through the sleep state. The ability of the SlS-GICA model to classify sleep states and extract spatiotemporal features and real-time analysis is one of the other advantages of the proposed model that has not been included in previous works.

Although the results of the current study demonstrate superior performance compared to the baseline systems in SlS classification, it does have certain limitations. Firstly, the EEG signals were recorded from only 21 channels in this study, which might not provide optimal spatial resolution. Increasing the number of channels would be advisable for better accuracy. However, it is important to consider that a large number of EEG channels during deep sleep could be cumbersome. Secondly, the dataset was recorded from subjects during a single session. To obtain more generalized attributes and assess the impact of music, it would be beneficial to repeat the experiment over a week. By using a subject-independent classification approach, our study aims to capture EEG patterns that are generalizable across subjects. This method enhances the robustness of the results, as the model’s performance is based on data from a variety of individuals, reducing the likelihood of overfitting to subject-specific characteristics. This is particularly relevant for applications seeking generalizable insights into cognitive load across diverse populations.

## 5 Conclusion

In this study, we presented a state-of-the-art approach for sleep state classification and investigation into the effect of auditory stimuli on REM sleep. The proposed method was based on functional connectivity and recurrence quantification analysis of pre-processed EEG signals. We recorded EEG signals from participants during wakefulness, NREM sleep, REM sleep with sound stimuli, and REM sleep without stimuli. The sound stimuli included 18 different types of sounds, such as musical instruments and nature sounds. After preprocessing the EEG signals, PLV, Coh, PAC, and RR were extracted from EEG sub-bands to obtain discriminative features and capture the complex behavior of the brain. The functional connectivity features (PLV, Coh, and PAC) were then used as inputs to a graph-informed convolutional autoencoder classifier. The graph-based approach plays a critical role in our method by capturing the underlying relationships between EEG channels that cannot be easily captured by standard statistical feature selection techniques alone. Instead of directly feeding the statistical features into the neural network, we extract graph-based features by analyzing the structure of this connectivity graph. These features provide a more comprehensive representation of the network dynamics, including higher-order interactions that are crucial to the complex relationships between brain regions. By using graph-based features, we ensure that the classifier is informed not only by traditional statistical measures but also by the spatial and functional relationships embedded in the graph. The attention layer also received the RR features to emphasize the dynamic analysis of the brain for each frame. Experimental results demonstrated that combining significant FC features with RR features in the attention layer of the GICA classifier leads to higher performance in sleep state classification, 99.92% in terms of accuracy (see Figure 12). Also, classifying the brain response to 18 different sound stimuli during REM sleep achieves an accuracy of 86.1% (see Figure 14 and Tables 4, 5).

The proposed SlS-GICA model has an important advantage over baseline methods. It performs sleep classification by utilizing the graphical representation of functional connectivity features and incorporates recurrence rate analysis in the attention layer of the deep convolutional classifier. Moreover, the classification performance indicates that the proposed SlS-GICA model outperforms recently published sleep state classification approaches by Mostafaei et al. (2024), Moctezuma et al. (2024), Li et al. (2022), Al-Salman et al. (2023), Eldele et al. (2021), and EEGNet (Lawhern et al., 2018), which serve as the baseline systems. Furthermore, the processing time window for EEG signals is generally substantial compared to previous studies. However, it is worth noting that the use of an attention-based convolutional autoencoder classifier has limitations in capturing long-term time series with high global dependencies.

The primary limitation of this study is that it was conducted on a cohort of 33 healthy subjects, without data from individuals with sleep disorders. Since sleep disturbances may alter EEG dynamics, the generalizability of our findings to clinical populations remains uncertain. Future research should extend this analysis to individuals with sleep disorders to validate the robustness of our approach and its potential clinical applications. Although the order of stimulus presentation was not randomized across participants, we minimized potential biases by maintaining identical conditions for all subjects. This approach ensured consistency in experimental exposure and facilitated direct comparisons across individuals. However, we acknowledge that a counter-balanced, within-subject design would be preferable in future studies to further mitigate potential order effects and strengthen the generalizability of our findings. A key avenue for future research is to validate the proposed model using a dataset of individuals with sleep disorders. This would be provided further insights into its diagnostic utility and potential for clinical application. The findings of this research have the potential to inform the development of interventions such as music therapy or low-frequency electrical stimulation of brain rhythms, which could potentially expedite the onset of sleep and alleviate symptoms associated with sleep disorders, such as difficulty falling asleep or insomnia.

## Data Availability

The original contributions presented in the study are included in the article/supplementary material, further inquiries can be directed to the corresponding authors.

## Footnotes

^1^https://www.nch.com.au/wavepad/index.html
