# Erratum: Graph-informed convolutional autoencoder to classify brain responses during sleep

**DOI:** 10.3389/fnins.2025.1627975

**Published:** 2025-05-28

**Authors:** Frontiers Production Office

**Affiliations:** Frontiers Media SA, Lausanne, Switzerland

**Keywords:** auditory stimuli, convolutional neural network, EEG, functional connectivity, graphical representation, sleep

Due to a production error, there was an error regarding the affiliation for Somayeh Makouei. Instead of having affiliation 2, they should have affiliation 1: “Faculty of Electrical and Computer Engineering, University of Tabriz, Tabriz, Iran”.

The publisher apologizes for this mistake. The original version of this article has been updated.

